# Genetic stability, genetic variation, and fitness performance of the genetic sexing Salaya1 strain for *Bactrocera dorsalis*, under long-term mass rearing conditions

**DOI:** 10.1186/s12863-020-00933-4

**Published:** 2020-12-18

**Authors:** Nidchaya Aketarawong, Siriwan Isasawin, Kamoltip Laohakieat, Sujinda Thanaphum

**Affiliations:** Regional R&D Training Center for Insect Biotechnology (RCIB), Department of Biotechnology, Faculty of Science, Mahidol University, Phayathai, Ratchathewee, Bangkok, 10400 Thailand

**Keywords:** Oriental fruit fly, Male recombination, Laboratory adaptation, SIT, Filter rearing system (FRS), Routine monitoring

## Abstract

**Background:**

A genetic sexing strain (GSS) is an essential component for pest control using the sterile insect technique (SIT). A GSS is developed using a combination of Y-autosome translocation and a selectable marker such as pupal color, resulting in heterozygous males and homozygous females that possess wild-type brown pupae (*wp*^+^) and mutant white pupae (*wp*) alleles, respectively. The genetic sexing Salaya1 strain developed for *Bactrocera dorsalis* was evaluated using a clean stream and scaled-up for subsequent production lines (e.g., initiation, injection, and release). Colony management under small- and large-scale conditions for long-term rearing may affect the sexing system, genetic background, and fitness performance of the strain. Routine monitoring was applied to study genetic stability, genetic variation, and male mating competitiveness.

**Results:**

The percentage of recombinants was significantly different between males (*wp*) and females (*wp*^+^), ranging between 0.21–0.43% and 0.01–0.04%, respectively. Using 106 bands from six ISSR markers, the genetic backgrounds of two generations (*F*_40_ and *F*_108_) of the clean stream were found to be almost identical (0.960), and between those two generations and the wild population, the similarities were 0.840 and 0.800, respectively. In addition, the sterile males performed well in competitive mating with fertile females (Relative Sterility Index = 0.67 ± 0.13). The rates of fliers calculated from both clean and release streams were higher than 0.95. Regarding the fitness of the Salaya1 strain, the fertility and pupal recovery were similar in all production lines. The sex ratio (Male/Female) distortion was also recorded.

**Conclusions:**

The Salaya1 strain reared at the mass-rearing facility retained its genetic stability, genetic variation, behavior (e.g., competitive mating and flight ability), and traits related to fitness for at least 10 consecutive generations. The filter rearing system is effective at minimising the selection pressure while maintaining the genetic background and fitness performances of the clean stream. These characteristics were stable throughout the production lines. In addition, the production efficiency is comparable among the different production lines and other similar types of GSSs.

**Supplementary Information:**

The online version contains supplementary material available at 10.1186/s12863-020-00933-4.

## Background

The sterile insect technique (SIT) is a widely accepted pest population suppression method due to its species specificity and environmentally-friendly nature. Pest-free areas can also be developed, maintained and protected by the integration of SIT [1]. The SIT program involves mass rearing and sterilisation by radiation of a target insect colony in a facility. Subsequently, the sterile insects are systematically released to compete against the wild male population. Hence, the reproductive potential of these wild males is reduced. One way to improve the effectiveness and cut operational costs of the SIT is to release high quality male-only sterile insects [2]. The release of sterile females is not desirable because ovipositing by pest damages fruits. The presence of sterile females also distracts the mating between the released sterile males and wild females during SIT application. The elimination of females lowers the cost of mass rearing and field release.

For tephritid fruit flies, the development of genetic sexing strains (GSSs) is made possible by the separation of the male and female individuals in the mass rearing. GSSs have been developed based on a classical genetic approach, which requires a combination of Y-autosome translocation (T(Y;A)) and a selectable marker (e.g. *white pupae* (*wp*), *black pupae* (*bp*), *temperature-sensitive lethal* (*tsl*) mutations) [2]. The Y-autosome translocation results in the linkage of Y chromosome and wild-type phenotype because they are heterozygous, one other allele carrying a wild-type allele on their Y-translocated chromosome and the other mutant allele on their free autosome. However, the females show a mutant phenotype because they are homozygous for the selectable marker. Vienna 7 [2], Vienna 8 [2], and Tapachula-7 [3] GSSs are examples of this type.

The genetic stability of the sexing phenotype is one of the major concerns in the quality management system during mass rearing. This is because recombination can occur between the chromosomal break point of the T(Y;A) and the selectable marker. Its frequency is lower if the autosomal break point is closer to the location of the selectable marker [2]. Rearing under stress conditions also increases the recombination frequency. The recombinant individuals, if they are not filtered out, they will accumulate. This leads to genetic breakdown and gradual loss of the sexing capacity in long-term mass rearing. The accumulation of recombinant individuals can be avoided by using a filter rearing system (FRS) [4–6].

The principle of FRS is to maintain a small standby GSS colony under minimal stress conditions (clean stream) where recombinants are physically selected out of the production. This clean stream must be cleaned off recombinants and be regularly used to refresh and/or continuously supply the mainstream of production lines with renewed genetic material. This can be accomplished using an amplification bridge that involves two steps, initiation and injection streams, which lead to the release stream. The initiation stream is used to increase the size of the clean stream, and the injection stream is used to inject new genetic material in to the release stream. Concurrently, the release stream is used to produce a large number of eggs and sorted male pupae for sterilisation and further field release [4]. The routine evaluation of genetic stability and biological quality of a GSS for different populations along the production streams incorporated in the FRS is essential for quality control in a mass-rearing facility. The insect quality can be used to predict the sterile male performance in the field. In addition, it can be used as a baseline to measure the effects of changes and scaling up in the production system. Furthermore, the data can be used to design, test, and improve rearing conditions [4–7].

Adaptation of the mass-reared GSS colonies is another important factors that affects the sustainability of SIT. The genetic response of the GSSs to environmental conditions may result in a deviation of genetic background and biological properties from their own ancestors, related strains, and wild populations [8]. Routine measurement of genetic diversity, mating competitiveness, traits related to fitness and rearing efficiency (fertility, egg-to-adult recovery rate, flight ability, and sex ratio) can be implemented for use in a quality control system. Although these parameters are specific to strain or mass-rearing protocol, they can be adopted as a standard protocol for GSS development and evaluation [7, 9–11].

The oriental fruit fly, *Bactrocera dorsalis*, is a key agricultural pest, causing reduction of fruit yield and limiting trade [12]. This fruit fly is native to far eastern Asia and was later distributed throughout Southeast Asia and the rest of Asia [13–15]. It has also spread outside its native range to some areas of the USA (i.e., California, Florida, and Hawaii), continental Africa (http://www.cabi.org/ISC/datasheet/17685; Accessed 26 May 2020), and recently, southern Italy [16]. The *B. dorsalis* Salaya1 GSS was developed based on the T(Y;A) translocation and *wp* gene. Their sex-linked traits produce brown-pupae males and white-pupae females [17]. The Salaya1 samples from *F*_5_ and *F*_17_ of the clean stream were evaluated for genetic variation. This strain was able to maintain genetic polymorphisms and mating competitiveness that were comparable to the wild target population. The Salaya1 strain was proven successful in a pilot-scale SIT project at suppressing the *B. dorsalis* wild population [17]. Currently, the clean stream is being reared for further amplification in a recently established mass-rearing facility.

In this study, we report on the genetic stability, genetic variation, mating competitiveness, and fitness parameters (e.g., fertility, egg-to-adult recovery rate, and flight ability) of the *B. dorsalis* Salaya1 strain in the continuous FRS for production. The Salaya1 strain shows low genetic recombination, indicating high genetic stability and the efficiency of the FRS. In addition, its genetic background and biological properties are stable under the long-term mass-rearing conditions. This suggests that the quality control data could be used as baselines for the mass rearing of the Salaya1 strain.

## Results

### Genetic stability

The genetic stability of the Salaya1 strain from clean, initiation, injection, and release streams were measured. Recombination occurring in the different streams was characterised as the accumulation of females emerging from brown pupae (*wp*^*+*^) (WT females) and males emerging from white pupae (*wp*) (*wp* males) under semi-mass rearing conditions over 10 consecutive generations (Table 1 and Additional file 1: Table S1). There was a significant difference between two types of recombinants (*t* = 6.094, *df* = 78, *P* < 0.001): The occurrence of WT females ranged from 0.01 to 0.04% while that of *wp* males ranged from 0.21 to 0.43%. Within the same type, the accumulated numbers of recombinants were not significantly different among the different streams (WT female: *F*_3,36_ = 0.843, *P* = 0.479; *wp* male: *F*_3,36_ = 0.693, *P* = 0.563).

**Table 1:** Genetic stability of the Salaya1 strain in the continuous filter rearing system for 10 consecutive generations

Colony	Adult emergence					Recombinant (%)	
	Brown pupae		White pupae		Total	WT female	***wp*** male
	Male	Female	Male	Female			
Clean stream	10,930	4	69	8410	19,413	0.02 a	0.36 b
Initiation stream	10,444	7	61	8009	18,521	0.04 a	0.33 b
Injection stream	10,546	2	40	8086	18,674	0.01 a	0.21 b
Release stream	7245	3	64	7422	14,734	0.02 a	0.43 b

The same letter is not significantly different from the others in the same type of recombinant (*P* < 0.05)

### Genetic variation

Genetic variation was evaluated in two generations (*F*_40_ and *F*_108_) of the clean stream and the wild population, using six inter simple sequence repeat (ISSR) markers. All 106 bands were used to estimate the number of alleles (Na), the number of effective alleles (Ne), expected heterozygosity (He), Shanon’s information index (I), and percentage of polymorphic loci (%P). The means of all the parameters estimated from the two generations of the clean stream, *F*_40_ (Na = 1.538; Ne = 1.427; He = 0.248; I = 0.371; %P = 70.75%) and *F*_108_ (Na = 1.387; Ne = 1.408; He = 0.236; I = 0.349; %P = 62.26%), showed slightly lower values than the wild population (Na = 1.726; Ne = 1.410; He = 0.258; I = 0.401; %P = 85.85%).

**Fig. 1:**
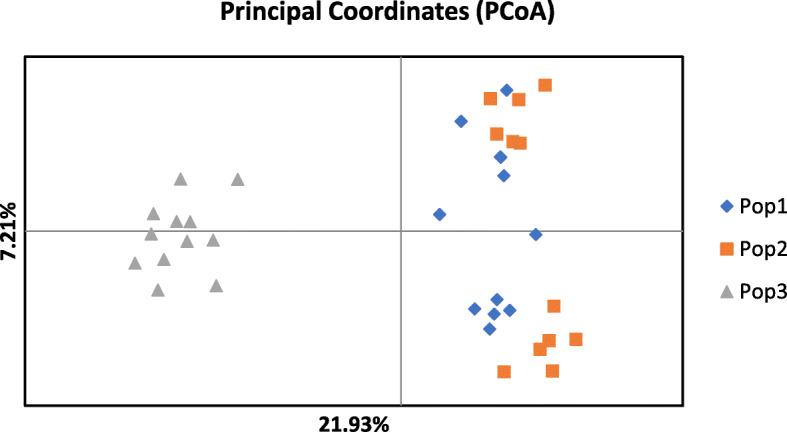
PCoA plot using a genetic distance matrix estimated with data from 36 samples. The planes of the first two principal coordinates explain 21.93 and 7.21% of total genetic variation, respectively. Pop1: the Salaya1 clean stream *F*_40_; Pop2: the Salaya1 clean stream *F*_108_; Pop3: the wild population

The Nei’s genetic identity of *F*_40_ and *F*_108_ were very close (0.960) as between those two generations and the wild population, 0.840 and 0.800, respectively. The Principle Coordinate Analysis (PCoA) plot using a genetic distance matrix illustrates the genetic divergence of fruit fly individuals from two generations of the clean stream and the wild population (Fig. 1). The first axis accounts for 21.93% of the total variation, which separates the two generations of the clean stream from the wild population while the second axis (7.21% of total variation) does not show any biological significance. The PCoA result is congruent with the genetic identity.

### Mating competitiveness of the Salaya1 strain

**Fig. 2:**
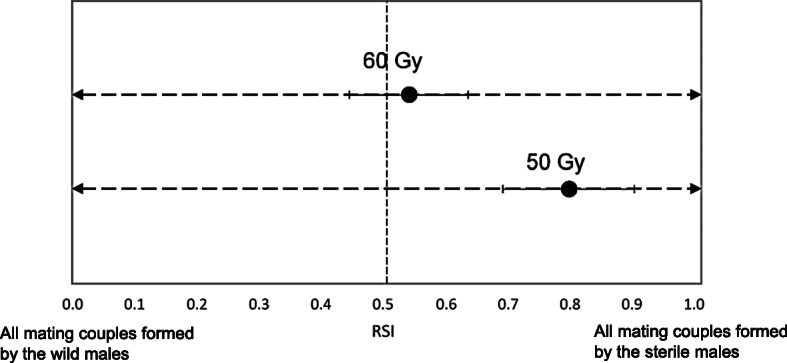
The relative sterility index (RSI) detected form the sterile male Salaya1 strain. RSI is estimated by the number of mating couples of the sterile males with wild females divided by the total number of mating couples [18]

The mating competition was observed between the sterile males of the Salaya1 release stream and wild males with wild females. Overall, the fruit flies used in mating performance evaluation were sexually mature (Proportion of mating (PM) ≥ 0.20). The mating analyses showed that the mean relative sterility index (RSI) was 0.80 ± 0.11 and 0.54 ± 0.10 when males of the Salaya1 strain were irradiated at 50 and 60 Gy, respectively (Fig. 2).

### Flight ability testing of the Salaya1 strain

Flight ability was evaluated with a standard protocol using adult emergence (%), adult fliers (%), and rate of fliers [18]. These values were compared between the clean and release streams for 10 consecutive generations (Additional file 2: Table S2). Although all of the observed values from the release stream were significantly lower than the clean stream (adult emergence: *t* = 4.521, *df* = 10.359, *P* = 0.001; adult fliers: *t* = 5.050, *df* = 10.127, *P* < 0.001; rate of fliers: *t* = 3.300, *df* = 18, *P* = 0.004), the averages of adult emergence and adult fliers were both well above 70%. The average rate of fliers was higher than 0.95 in both streams.

### Traits related to fitness within the Salaya1 and wild-type strains

Fertility, pupal recovery, adult emergence, and sex ratio were estimated from the *B. dorsalis* wild-type Phayathai1 strain and the production lines (e.g., clean, initiation, and injection streams) of the Salaya1 strain through 10 consecutive generations (Additional file 3: Table S3). The fertility (%) was similar in the different production lines of the Salaya1 strain but was significantly lower than in the Phayathai1 strain (*F*_3,36_ = 484.459, *P* < 0.001). The mean fertility in the Salaya1 strain, ranging from 47.06 ± 1.24% to 49.41 ± 0.80% among different streams, was approximately half of the value observed in the Phayathai1 strain (88.63 ± 0.47%). The pupal recovery rate (%) also indicated a similar trend (*F*_3,36_ = 33.559, *P* < 0.001). The mean pupation value of the Salaya1 strain varied from 75.78 ± 0.98% to 77.20 ± 1.17% while that of the Phayathai1 strain was 89.25 ± 1.20%.

The rates of adult emergence (%) were similar between strains and among production streams (*F*_3,36_ = 2.272, *P* = 0.097). The mean sex ratio between males and females observed in the Phayathai1 strain was close to 1:1 but was significantly different from the clean, initiation, and injection streams of the Salaya1 strain (*F*_3,36_ = 41.461, *P* < 0.001), for which the sex ratios were close to 1.3:1 (Additional file 3: Table S3).

The mean of egg-to-adult recovery rate in the Salaya1 strain, ranging from 26.73 ± 3.11 to 27.48 ± 2.14 among different streams, was significantly lower than in the Phayathai1 strain (61.29 ± 1.24) (*F*_3,36_ = 321.662, *P* < 0.001) (Additional file 3: Table S3).

### Production efficiency of the Salaya1 strain under semi-mass rearing conditions

Egg and pupae production, pupa weight, and egg-to-adult recovery rate were estimated from different streams of the Salaya1 strain over 10 consecutive generations (Additional file 3: Table S3). The clean stream had significantly higher mean egg production (eggs/female/day) than the initiation, injection, and release streams (*F*_3,32_ = 8.743, *P* < 0.001). The three latter production lines produced means for eggs/female/day of similar values, ranging from 23.18 ± 2.44 to 27.67 ± 3.34. However, the pupae production and egg-to-adult recovery rate were not significantly different among various streams (pupae production: *F*_2,27_ = 0.36, *P* = 0.791; egg-to-adult recovery rate: *F*_2,27_ = 0.237, *P* = 0.791). The pupa weight of each stream was approximately 11 to 12 mg for both brown and white pupae, which is comparable to the pupa weight of the wild-type Phayathai1 strain.

## Discussion

### Genetic stability of the Salaya1 strain

**Fig. 3:**
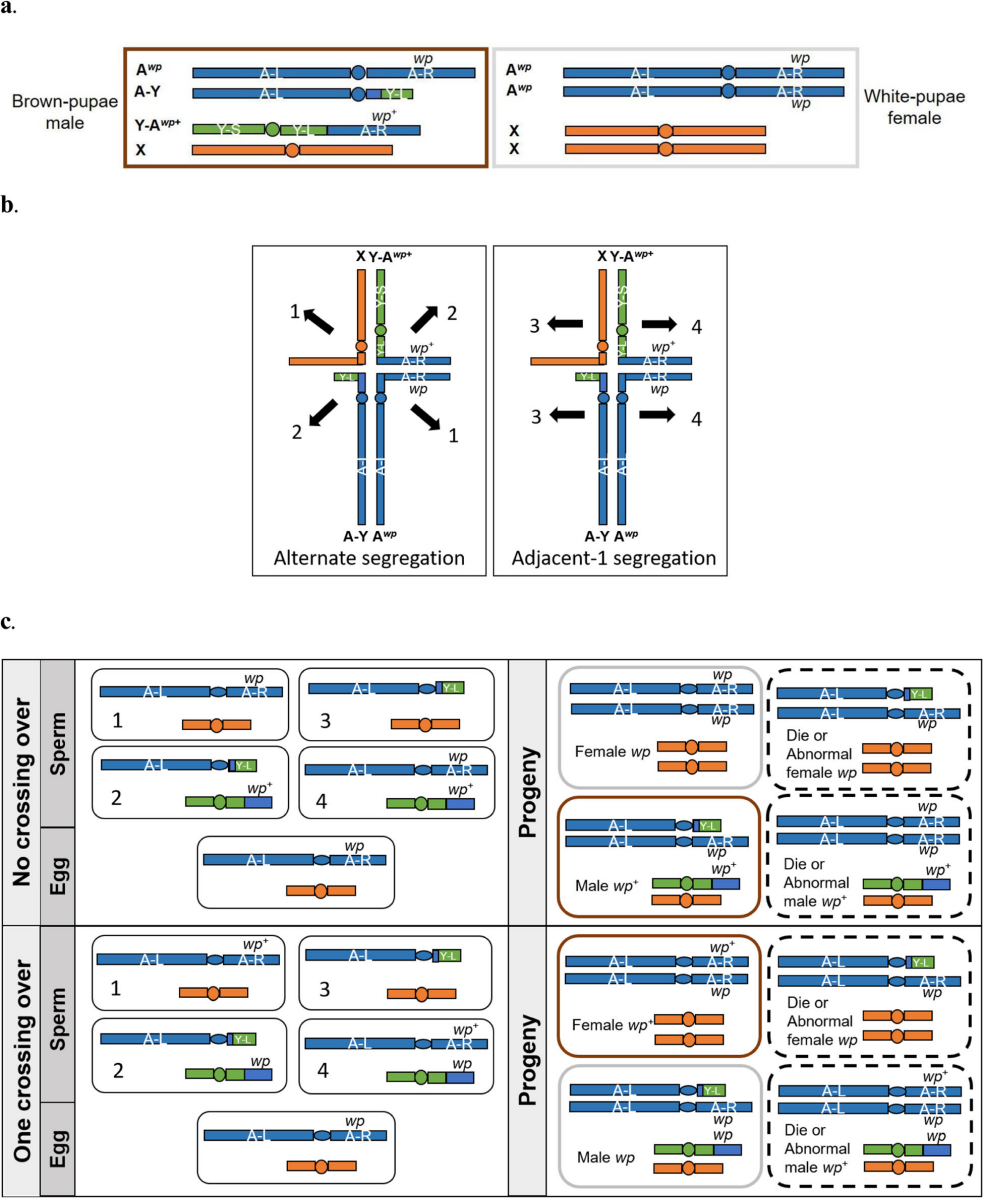
Schematic representation of a basic Y-autosome translocation and putative consequence in the Salaya1 strain. **a** A combination of Y-autosome translocation and a selectable marker of pupal color; *wp +* and *wp* represent the wild-type allele (brown pupal color) and the mutant allele (white pupal color), respectively. The two reciprocal components of the Y-autosome translocation are Y-A^*wp+*^ and A-Y. **b** The two types of segregation of Y-autosome translocation during male meiosis: alternate and adjacent-1 (modified from [2]). **c** The mating schemes with no and one crossing over. No crossing over produces 50% of genetically normal progenies (e.g., brown-pupae males (brown border) and white-pupae females (gray border) whereas the rest of the progenies are autosomal deletion or triplication types (dash border). On the other hand, one crossing over presents 50% of sex-reversal progenies (e.g., brown-pupae females (*wp+*) and white-pupae males (*wp*)) whereas the rest are aneuploidy. Y-A^*wp+*^: translocation fragment carrying a Y chromosomal centromere; A-Y: reciprocal translocation fragment carrying an autosomal centromere; X: X chromosome; and A^*wp*^: autosome

During long-term strain management, the sexing system may be lost, which causes strain breakdown, mostly either due to the recombination in the chromosome region between the translocation break point and the selectable markers (e.g., *wp*) or the survival of adjacent-1 segregates [2, 19]. Accumulation and selection in favor of recombinants can occur rapidly and depend on the environmental stress conditions [4, 19]. Monitoring for stability of the sexing system is necessary to ensure their positive characteristics [20]. Firstly, a GSS must present a relatively low level of recombination due to its short distance between the break point and the selectable markers of the T(Y:A). Secondly, a GSS should produce no or few progenies related to adjacent-1 segregation during male meiosis (Fig. 3b). The progenies with autosomal deficiency usually die at the embryonic stage while the triplication type adjacent-1 progenies may be viable through to the adult stage [21]. Lastly, a GSS should have only one T(Y;A) break point. Higher complexity of chromosomal rearrangement including a potential multiple translocation system could lead to the generation of more unbalanced gametes (autosomal deficiency or duplication of chromosomal regions) of the translocation heterozygotes. This situation would increase the sterility level of the strain [19, 21, 22]. The genetic sexing Salaya1 strain for *B. dorsalis* seems to be stable. The number of accumulated recombinants was relatively low in both types of recombinants: females in brown pupae (WT female; 0.02%) and males in white pupae (*wp* male; 0.36%). This suggests that the T(Y:A) break point is closely linked to the *wp* locus. Different ratios between the two types of recombinants were also observed in the other GSSs (e.g., *C. capitata* [19] and *Z. cucurbitae* [23]). According to the potential outcome of segregation and crossing over during male meiosis (Fig. 3b and c), the two types of recombinants should occur at an equal ratio. The reliance on only pupae color sorting may sometimes cause results that are not as sufficiently accurate as the results found for other GSSs, such as in the case of *C. capitata* [7], *Z. cucurbitae* [23], and *A. ludens* [3, 24]. The puparium depigmentation in the wild-type pupae can be affected by nutritional deficiency [25, 26]. The sorted brown pupae females of the Salaya1 occur at very much lower rate (0.02%). Hence, they affect very little in the male-only release system for SIT. In addition, the numbers of *wp* male recombinants did not significantly accumulate in all production lines, even during the 10 generations of circulating the release stream. In this study, the low recombination rate suggested that the FRS is practical and effective for minimising the genetic instability. In addition, the egg-to-adult recovery rate of the Salaya1 strain (27.48%) was significantly different from its ancestral wild-type Phayathai1 strain (61.29%) by roughly 50%. This 50% reduction is consistent with the theoretical expectation that the Salaya1 possesses a simple T(Y;A) translocation which is suitable for mass-rearing production [3, 22, 24, 27]. When a simple Y-autosome translocation involving only one autosome was cytogenetically confirmed in the other GSS of *B. dorsalis*, the egg-to-adult recovery rate was also approximately 50% [21].

### Standards for the maintenance of genetic integrity in the Salaya1 strain

A set of characteristics that are unique to the DNA of populations are the chief distinguishing aspects of the genetic profile of GSSs. The integrity of a shared genetic profile can be lost during strain improvement and colony management. This could affect the mass-rearing efficiency and field performance of the sterile males. The same GSS may manifest varied biological properties (e.g., fertility, eggs to pupae rate, and adult emergence) when reared in different places and/or conditions [7]. This situation has been generally observed in several established GSSs such as the Vienna 7 and Vienna 8 strains of *C. capitata* [7, 11], the 49-wp strain of *B. dorsalis* [28], and the species *Z. cucurbitae* [23]. In this study, small samples of fruit flies were evaluated under laboratory conditions. In the scaled-up rearing, it was found that the biological properties were similar in all production lines. The acquired data could be used as reference for the Salaya1 strain. The data will allow standard comparison among Salaya1 colonies from different time points, production levels, and mass-rearing conditions. In doing so, the standards of biological quality control should include the rate of egg hatch, pupation, adult emergence, egg-to-adult recovery rate, and recombinants.

Maintenance of the genetic integrity is a primary consideration during the colonisation stage and in mass-rearing management of the strain. The heterozygosity can be affected by the genetic diversity of founding individuals and the degree of inbreeding in production lines [29]. A sufficient number of rearing individuals is therefore an important factor for sustaining genetic variation, mass rearing efficiency, and field performance of the sterile males. Under the current quality management system, the Salaya1 strain has retained its genetic integrity for 10 years when compared to the wild population. It has been maintained at the level of approximately 5000 individuals per generation. Isasawin and colleagues [17] and this study tested the genetic background of the different generations of the Salaya1 clean stream and wild population using microsatellite and ISSR markers, respectively. Even though the ISSR markers are dominant markers, a sufficient number of marker bands (depending on analysis method, e.g., a modest number of 30–50 bands for AMOVA and generally 90 or more for STRUCTURE) and individuals (more than 10) could provide satisfactory results [30]. The results from 106 ISSR bands were similar to those from four polymorphic microsatellite loci. However, the ISSR marker system required less time and expense, as well as provided data as DNA fingerprinting. DNA banding patterns of ISSR_02 to 05 are examples of markers for primary screening of genetic background in the colony (Additional file 4: Fig. S1 to S6). Therefore, the ISSR system may be a potential tool for large-scale monitoring of genetic variation in a mass-rearing facility [31].

### Potential for improvement of Salaya1 mass rearing

In this study, we present the minimum viable production for mass rearing of the Salaya1 strain, at least for maintaining the main production via amplification bridge (initiation and injection streams) at regular intervals [4]. However, the strain fertility can be improved to increase egg-to-adult recovery rate at an industrial scale. Eggs can be initially incubated at a constant temperature using a bubbling system (e.g., an air pump for a fish tank) before seeding on a larval diet [32–34]. This step promotes embryonic development by providing higher oxygen levels. In order to enhance a high number of larvae production, a greater number of seeding eggs placed into the larval food can compensate for the low fertility rate [28, 35]. However, to offset production loss, a surplus of seed eggs should be avoided as this caused diminishing return. In addition, characterisation of the strain using cytogenetic analyses can provide more accurate information on the structure of the chromosome rearrangement and mapping of the chromosome break point [3, 28]. Introducing chromosomal inversion on the autosome carrying the selectable markers can cause a recombination-reducing effect [2, 27]. This is exemplified in the case of *C. capitata*, in which an autosomal inversion had been introduced to a translocation T(Y;5)101 line that was not very stable [2]. When the autosomal inversion carrying the selectable markers (*wp* and *tsl*) was introduced, the stability was very high. This led to a new strain, called Vienna 8^D53+^ which was later adopted in several mass-rearing facilities [2, 7, 27].

To prevent possible loss of genetic variation during colonisation of GSSs, new genetic material can be sometimes introduced by outcrossing to wild flies from the target population [36] or by inter-crossing between independent domesticated lines [37]. This could maintain the level of genetic variation, as well as improve the rearing efficiency and field performance of the genetically refreshed strain [37].

Mating competitiveness data of the Salaya1 strain suggested that it has equal mating performance to the wild males when they were sterilised with a sterilisation dose of 60 Gy. This was estimated by the RSI value of 0.54 ± 0.10. A much higher value of RSI (0.80 ± 0.11) demonstrated that the Salaya1 sterile males can succeed more often in the mating competition with the wild females when they were irradiated at 50 Gy. This RSI value is unusally high when compared with the values from GSSs Vienna 8 (0.22 to 0.34) [38, 39] and Tapachula-7 (0.4) [40]. However, this is consistent with a previous finding. With a 50 Gy sterility dosage, the Salaya1 sterile males could generate 50% sterility in the wild females in a similar mating competition scheme [17]. It was found that more wild females were attracted to the sterile males than the wild males of *C. capitata* in field cages [41]. The sterile males showed more extensive calling behavior than the wild males [41]. Comparative study of the mating behavior between the Salaya1 strain and the wild types should be carried out to improve the SIT. The results stemming from different sterilising doses (50 versus 60 Gy) suggest that the small radiation dosages utilised in this experiment can strongly relate to the mating competitiveness of the males. Radiation dosimetry and sterilising dose should be critically studied to maximise the mating competitiveness of the Salaya1 sterile males.

## Conclusions

The Salaya1 strain, reared in both relaxed conditions and high-density rearing conditions at the mass-rearing facility, can retain its genetic stability, genetic variation, rearing efficiency, and field performance (e.g., competitive mating and flight ability). The stable parameters imply that the FRS implemented in this study is suitable. Incorporation of an autosomal inversion and additional selectable marker lines to the Salaya1 are desirable to refine the strain. The Salaya1 strain improvement can be evaluated based on the standard monitoring of the FRS system suggested in this work. According to the consistency of rearing profiles throughout different production lines, the Salaya1 strain is comparable to other pupae-color based GSSs and is ready for industrial-scale rearing and SIT programs.

## Methods

### Fruit fly strains

The Salaya1 strain is a GSS based on brown-white pupal colour dimorphisms for sex sorting [17]. This strain had been developed and continuously maintained at a laboratory-scale and modular mass-rearing scale as a clean stream since 2009. In 2016, the standard mass-rearing facility named ‘Regional R&D Training Center for Insect Biotechnology-RCIB’ was launched. The initiation stream was initially colonised. Two years later, the injection and release streams were established and used in this study.

The Phayathai1 strain is a wild-type bisexual strain and also the original strain used for development of the Salaya1 strain [17]. This strain was also used as a reference strain for studying genetic variation [42, 43] and has been continuously maintained under the laboratory conditions for more than 15 years.

Wild samples were obtained from larvae and eggs collected from infested organic orchards of mixed fruits (e.g., guava, mango, rose apple, and banana) in Nakhon Pathom Province, Thailand. These samples were reared on their host fruits in the quarantine section at the RCIB. The flies from the *F*_1_ generation were used for genetic variation tests while the rest of the samples were used to generate the further generations. Only the *F*_2_-*F*_4_ generations were used for the mating competitiveness experiments to ensure wild samples still maintained their wild performance equivalency [44].

### Laboratory and semi-mass rearing conditions

**Fig. 4:**
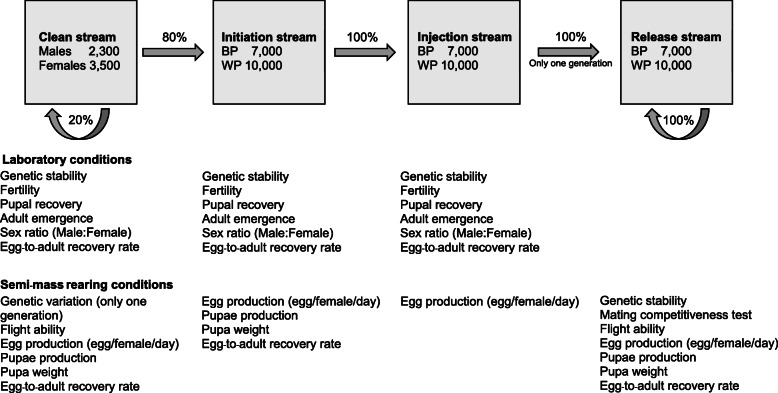
Schematic flow chart of the Salaya1 production lines. Each stream is monitored and compared to the other streams. The meaning of each parameter is described in the text

The wild-type Phayathai1 strain has been continuously reared in a laboratory cage with the dimensions of 0.35 × 0.45 × 0.35 m (width x length x height). Every generation, approximately 600 adult flies are used to maintain their colony. On the other hand, at a semi-mass rearing scale, the Salaya1 strain has been continuously reared in a mass-rearing cage with the dimensions of 0.4 × 1.0 × 1.8 m (width x length x height). Every generation, approximately 6000, 17,000, and 17,000 adults are used to establish their clean, initiation, and injection streams, respectively (Fig. 4). Only one generation of the injection stream was used for colonisation of a release stream, and the release stream was maintained similar to the initiation and injection streams. All strains and streams have been maintained under a photoperiod of 13:11 [L:D] h at 25–28 °C with 70–80% RH. The adult diet contains a mixture of hydrolysate yeast and sugar in a ratio of 1:3 by weight. Water is supplied to the cages by polypropylene cup with a cotton wick, and a bottle placed over a paper towel.

After 14–15 days of adult emergence, small perforated plastic tubes and bottles painted inside with guava juice were placed into the laboratory and mass-rearing cages, respectively, for egg collection. These eggs were seeded on a standard larval semisolid diet (26% wheat bran, 12% sugar, 3.6% hydrolysate yeast, 0.1% sodium benzoate, 0.1% methyl-p-hydroxyl benzoate, 0.2% acetic acid and 58% water by weight) [45]. Eight to ten days after egg collection, mature larvae popped out of the food and formed pupae in the sawdust. All strains were parallel-reared using the same protocol. The Phayathai1 strain and Salaya1 clean stream were maintained in the standard insectary room (96 m^2^) while the Salaya1 initiation, injection, and release streams were maintained at the standard mass-rearing facility (697 m^2^).

Both strains and each stream were maintained over 10 consecutive generations for this study. The life cycle of each generation was approximately 1 month.

### Genetic stability

The presence of recombinants was evaluated by separating white and brown pupae and adults according to their sex. Materials used were the same as those from the section of fitness measurement (mentioned later). The most frequent recombination of the genetic sexing strain takes place between the translocation break point and *wp* allele (type 1) [9]. This phenomenon produces males emerging from white pupae (*wp*) and females emerging from brown pupae (*wp*^+^). Observed recombinants from the type 1 recombination was estimated in each stream at three to five replications per generation for 10 consecutive generations. One-way ANOVA was used for the comparisons among different streams. Tukey’s HSD test was performed as a post hoc test for all pairwise comparisons. The statistical program PASW statistics 18 was employed for the analysis [46].

In addition, the rate of recombinants was assessed in 10 consecutive release stream generations. One thousand brown and white pupae were randomly collected from each generation, and emerged adults were observed according to their sex.

### Genetic variation

Twelve fruit fly samples from the Salaya1 clean stream (*F*_40_ and *F*_108_) and the wild population were randomly selected. Genomic DNA was individually extracted from adult fruit flies as per Baruffi et al. 1995 [47]. The ISSR markers were used to test genetic variation of all samples because ISSR can generate a multiple banding pattern by amplification of the regions between microsatellites [31, 48, 49]. Six primers containing core microsatellite and anchor sequences and their selected bands are described in Additional file 5: Table S4. Each PCR was carried in a 20 μl volume containing 10 ng of genomic DNA, 1x buffer, 1.5 mM MgCl_2_, 0.2 mM dNTPs, 1 U *Taq* polymerase (Vivantis), and 40 pmol primer. PCR conditions used are as follows: 94 °C for 5 min; 3 cycles of 94 °C for 30 s, 58 °C for 1 min, 72 °C for 1 min; 3 cycles of 94 °C for 30 s, 56 °C for 1 min, 72 °C for 1 min; 3 cycles of 94 °C for 30 s, 54 °C for 1 min, 72 °C for 1 min; 30 cycles of 94 °C for 30 s, 52 °C for 1 min, 72 °C for 1 min; 72 °C and one cycle of final extension at 72 °C for 5 min.

PCR products were evaluated using 2% agarose gel at 50–70 V for 3–5 h, compared to 100 bp plus DNA ladder (Thermo Fisher Scientific). Gel contained 0.5 μmol ethidium bromide while the solution in the chamber contained 1X TBE buffer and 0.5 μmol ethidium bromide.

The presence and absence of each ISSR-PCR amplicon were scored as ‘1’ and ‘0’, respectively. Multiple ISSR-PCR banding patterns derived from different ISSR primers could be used together to create a 1/0 binary genotypic profile for each sample (Additional file 6: Data sheet). These diploid binary profiles were used as input data for GenAlEx v.6.503 using the Hardy-Weinberg equilibrium [50]. The number of alleles (Na), the number of effective alleles (Ne), expected and unbiased expected heterozygosity (He and uHe, respectively), Shanon’s information index (I), Percentage of polymorphic loci (%P), and Nei’s genetic identity were estimated. PCoA was analysed, and a two-dimensional graph was plotted using a subprogram in GenAlEx [50].

### Measurements of strain performances

#### Mating competitiveness test

The experiments were carried out according to the standard protocol from FAO/IAEA/USDA [18]. This was done in order to observe the ability of sterile males of the Salaya1 strain to compete with fertile wild males for mating with fertile wild females. The brown pupae from the release streams (*F*_7_-*F*_9_) were sterilised with a radiation dose of 50 and 60 Gy (MultiRad160, Faxitron) during their late pupal stage (2 days before emergence). The irradiation doses of 50 and 60 Gy were chosen because they provided 100% male sterility when approximately 2000 eggs were tested in three replications. Eye color change was used in parallel to assure the development of pupae. Emerged sterile males were maintained under a photoperiod of 13:11 [L:D] h at 25–28 °C with the adult diet and the displayed courtship behavior (e.g., male calling) was observed to ensure the sexual maturity. The tested wild flies were sex sorted within 1 day of emergence and similarly reared. All flies were maintained in the same manner as sterile males. At least 4 h before the mating experiment, the sterile and wild males were spot-marked with different water-based colors (white and gray, respectively) at their dorsal thoraxes. Rotation of the water-based dye marking between the sterile and wild males was not carried out due to limitations on the availability of the wild samples. One possible concern is that the colour could have an effect on the mating choices made by females, however, there have been no such observations from similar experiments [38, 39]. The study was conducted in three outdoor field cages 3 × 3.5 × 2.3 m (width x length x height) with a potted mango tree inside each. In each mating competitiveness test, 20 sterile males and 20 wild males were released 30 min earlier to allow lekking formation in the cages. Twenty wild females were subsequently released at 17:00. Only 20 wild males and 20 wild females were released in the control cages. Mating pairs were observed between 18.00–19.30. The mating couples of either sterile or wild males to the wild females were individually collected in plastic vials and counted.

Data from the control cage was used to calculate the proportion of mating (PM) [18]. This parameter ensures the readiness of the flies and adequacy of environmental conditions for the mating test. The PM value was calculated by the number of mating pairs divided by the total number of released females. If the PM value was less than 0.2, the experiments were discarded. The relative sterility index (RSI) was calculated to determine the male sexual competitiveness. The values of RSI range from 0 (i.e., all of wild females mate with wild males) to 1 (i.e., all wild females mate with sterile males). A value of 0.5 indicates an equal mating performance ratio for sterile and wild males. The mating competitiveness experiment was repeated in triplicate, and the RSI values were compared using the Student’s *t*-test in PASW statistics 18 [46].

#### Flight ability testing

The experiments were carried out to estimate the ability of the brown pupae to emerge and develop into flying male adults, according to the standard protocol from FAO/IAEA/USDA [18]. The brown pupae samples were taken from the clean and release streams. The experiment was performed in a flight testing apparatus; (i.e., black-painted plexiglass tube: outside diameter 8.9 cm with 3 mm wall thickness and 10.0 cm height). One hundred pupae were loaded into a closed flight testing apparatus approximately 2 days before their expected emergence. The pupae-loaded flight testing apparatus was placed in a transparent plastic container. The lid of the apparatus had been opened before the plastic container was closed to allow the adults to fly out. There are five outcome categories; non-emerged pupae, partially-emerged pupae, deformed adults, non-flier adults, and flier adults. The data from these categories were recorded daily and summarised after a period of 7 days.

The percentage of adult emergence was calculated by the total number of fully-emerged flies (i.e., deformed flies, non-fliers, and fliers) divided by the total number of pupae whereas the percentage of fliers was calculated from the number of fliers divided by the total number of pupae. The rate of fliers equals the percentage of fliers divided by the percentage of emergence [18]. The flight ability test was done in five replicates. The Student’s *t*-test was used for the comparisons among different streams using PASW statistics 18 [46].

### Measurements of traits related to fitness and production efficiency of the strain

#### Fitness measurement

Samples were randomly selected, observed, and recorded at all stages of development. The quality control parameters, including fertility (egg-hatching rate), egg to pupae recovery (pupal recovery rate), pupa weight, adult emergence, and sex ratio of the Salaya1 strain, were tested following the standard protocol [18].

Fertility was evaluated from 1400 eggs per replicate. The eggs were aligned on a piece of wet paper and placed on larval food in a small cup. Eggs were incubated at 25–28 °C for 3 days, and the egg-hatching rate was determined. This standard was followed for every generation of both strains and each stream collected in three to five replicates for 10 consecutive generations.

Pupal recovery was calculated by the number of pupae divided by the number of hatched eggs. Pupa weight was calculated by measuring the weight of 20 randomly selected pupae. Three replicates per generation were performed and adult emergence was evaluated by the number of normal adults divided by the number of pupae. The sex ratio of males to females was also calculated.

Egg-to-adult recovery rate was calculated to evaluate the production efficiency. This parameter was estimated by the number of normal adults divided by the number of reared eggs.

One-way ANOVA was used for the comparisons among different strains and streams. Tukey’s HSD test was performed as a post hoc test for all pairwise comparisons. The statistical program PASW statistics 18 was employed for the analysis [46].

#### Production efficiency

Egg production was evaluated by the number of eggs produced per female per day. The number of eggs in 1.0 mL laid on the wet paper were counted three times, and the mean value was 17,500 ± 2% eggs/ml. This value was used to multiply with the total volume of eggs collected daily and divided by 74% of the number of white pupae for calculation of the egg production. This is because approximately 74% of pupae were emerged, and only females emerged from white pupae. Under the semi-mass rearing conditions, eggs were reared on larval food in a tray, and the fertility was not monitored. Pupae production was used instead of pupal recovery and was evaluated by the number of pupae divided by the number of reared eggs.

Egg-to-adult recovery rate was calculated to evaluate the production efficiency at the mass-rearing scale when all processes were done in the facility. This parameter was estimated by the number of normal adults divided by the number of reared eggs. The number for adult emergence was estimated from the number of pupae multiplied by 74%. One-way ANOVA was used for the comparisons among streams. Tukey’s HSD test was performed as a post hoc test for all pairwise comparisons. The statistical program PASW statistics 18 was employed for the analysis [46].

## Supplementary Information

**Additional file 1:** **Table S1.** Genetic stability of the Salaya1 strain in the continuous filter rearing system under semi-mass rearing conditions.

**Additional file 2:** **Table S2.** Flight ability (mean ± standard error) of the Salaya1 clean and release streams.

**Additional file 3:** **Table S3.** Production efficiency of the Salaya1 strain in each production line under laboratory and semi-mass rearing conditions.

**Additional file 4:** **Figure S1.** Results of ISSR_01 marker analysis. **Figure S2.** Results of ISSR_02 marker analysis. **Figure S3.** Results of ISSR_03 marker analysis. **Figure S4.** Results of ISSR_04 marker analysis. **Figure S5.** Results of ISSR_05 marker analysis. **Figure S6.** Results of ISSR_06 marker analysis.

**Additional file 5:** **Table S4.** Six polymorphic ISSR primers and their matrices of obtained band sizes.

**Additional file 6.** Data sheet.

**Additional file 7:** **Figure S7.** Raw gel images from ISSR_01 marker analysis. **Figure S8.** Raw gel images from ISSR_02 marker analysis. **Figure S9.** Raw gel images from ISSR_03 marker analysis. **Figure S10.** Raw gel images from ISSR_04 marker analysis. **Figure S11.** Raw gel images from ISSR_05 marker analysis. **Figure S12.** Raw gel images from ISSR_06 marker analysis.

## Data Availability

All supporting data are included as additional information.

## Abbreviations

ANOVA: Analyses of Variance
FRS: Filter Rearing System
dNTPs: Deoxynucleotide triphosphates
GSS: Genetic sexing strain
He: Expected heterozygosity
I: Shanon’s information index
ISSR: Inter simple sequence repeat
Na: Number of alleles
Ne: Number of effective alleles
PCR: Polymerase chain reaction
PCoA: Principle Coordinate Analysis
%P: Percentage of polymorphic loci
PM: Proportion of mating
RSI: Relative sterility index
SIT: Sterile insect technique
uHe: Unbiased expected heterozygosity
