# Supplementary material for: Genetic stability, genetic variation, and fitness performance of the genetic sexing Salaya1 strain for Bactrocera dorsalis, under long-term mass rearing conditions
Source: BMC Genet. 2020 Dec 18;21(Suppl 2):131. doi: 10.1186/s12863-020-00933-4 (PMC7747453; doi:10.1186/s12863-020-00933-4)
Supplement: Supplementary file 1 — Additional file 1: Table S1. Genetic stability of the Salaya1 strain in the continuous filter rearing system under semi-mass rearing conditions. [file 12863_2020_933_MOESM1_ESM.pdf]

Additional file 1:

Table S1. Genetic stability of the Salaya1 strain in the continuous filter rearing system under semi-mass rearing conditions

**Colony**	**Gen**	**Adult emergence**					**Recombinant (%)**	
		**Brown pupae**		**White pupae**		**Total**	**WT female**	***wp* male**
		**Male**	**Female**	**Male**	**Female**			
**Clean stream**	1	901	0	19	696	1,616	0.00	1.18
	2	815	0	19	601	1,435	0.00	1.32
	3	1,064	0	3	837	1,904	0.00	0.16
	4	1,098	1	3	901	2,003	0.05	0.15
	5	1,181	0	1	922	2,104	0.00	0.05
	6	1,165	1	7	796	1,969	0.05	0.36
	7	1,232	0	4	938	2,174	0.00	0.18
	8	1,185	2	11	909	2,107	0.09	0.52
	9	1,174	0	1	933	2,108	0.00	0.05
	10	1,115	0	1	877	1,993	0.00	0.05

Table S1. Genetic stability of the Salaya1 strain in the continuous filter rearing system under semi-mass rearing conditions (cont.)

**Colony**	**Gen**	**Adult emergence**					**Recombinant (%)**	
		**Brown pupae**		**White pupae**		**Total**	**WT female**	***wp* male**
		**Male**	**Female**	**Male**	**Female**			
**Initiation stream**	1	852	0	10	668	1,530	0.00	0.65
	2	883	2	13	689	1,587	0.13	0.82
	3	1,078	0	5	783	1,866	0.00	0.27
	4	1,125	0	9	865	1,999	0.00	0.45
	5	858	1	1	712	1,572	0.06	0.06
	6	1,092	0	9	776	1,877	0.00	0.48
	7	1,196	0	0	847	2,043	0.00	0.00
	8	1,085	3	10	861	1,959	0.15	0.51
	9	1,269	1	3	977	2,250	0.04	0.13
	10	1,006	0	1	831	1,838	0.00	0.05

Table S1. Genetic stability of the Salaya1 strain in the continuous filter rearing system under semi-mass rearing conditions (cont.)

Colony	Gen	Adult emergence					Recombinant (%)	
		Brown pupae		White pupae		Total	WT female	*wp* male
		Male	Female	Male	Female			
Injection stream	1	763	0	9	545	1,317	0.00	0.68
	2	859	0	4	598	1,461	0.00	0.27
	3	1,017	0	2	729	1,748	0.00	0.11
	4	1,151	1	2	849	2,003	0.05	0.10
	5	1,063	0	1	915	1,979	0.00	0.05
	6	1,154	0	13	894	2,061	0.00	0.63
	7	1,181	0	3	886	2,060	0.00	0.14
	8	1,152	0	2	936	2,090	0.00	0.10
	9	1,132	1	1	855	1,989	0.05	0.05
	10	1,074	0	3	879	1,956	0.00	0.15

Table S1. Genetic stability of the Salaya1 strain in the continuous filter rearing system under semi-mass rearing conditions (cont.)

Colony	Gen	Adult emergence					Recombinant (%)	
		Brown pupae		White pupae		Total	WT female	*wp* male
		Male	Female	Male	Female			
Release stream	1	830	2	9	799	1,640	0.12	0.55
	2	695	0	14	638	1,347	0.00	1.04
	3	770	0	13	798	1,581	0.00	0.82
	4	663	0	8	572	1,243	0.00	0.64
	5	808	0	8	889	1,705	0.00	0.47
	6	654	0	1	693	1,348	0.00	0.07
	7	752	1	4	726	1,483	0.07	0.27
	8	616	0	1	728	1,345	0.00	0.07
	9	717	0	1	778	1,496	0.00	0.07
	10	740	0	5	801	1,546	0.00	0.32
