# Supplementary material for: Genetic stability, genetic variation, and fitness performance of the genetic sexing Salaya1 strain for Bactrocera dorsalis, under long-term mass rearing conditions
Source: BMC Genet. 2020 Dec 18;21(Suppl 2):131. doi: 10.1186/s12863-020-00933-4 (PMC7747453; doi:10.1186/s12863-020-00933-4)
Supplement: Supplementary file 2 — Additional file 2: Table S2. Flight ability (mean ± standard error) of the Salaya1 clean and release streams. [file 12863_2020_933_MOESM2_ESM.pdf]

Additional file 2:

Table S2. Flight ability (mean ± standard error) of the Salaya1 clean and release streams

	Clean stream	Release stream
Adult emergence (%)	86.42 ± 0.73 a	74.06 ± 2.64 b
Adult fliers (%)	85.06 ± 0.66 a	71.30 ± 2.64 b
Rate of fliers	0.98 ± 0.01 a	0.96 ± 0.02 b

The same letter is not significantly different from the other in the same parameter ($P < 0.05$).
