# Supplementary material for: Genetic stability, genetic variation, and fitness performance of the genetic sexing Salaya1 strain for Bactrocera dorsalis, under long-term mass rearing conditions
Source: BMC Genet. 2020 Dec 18;21(Suppl 2):131. doi: 10.1186/s12863-020-00933-4 (PMC7747453; doi:10.1186/s12863-020-00933-4)
Supplement: Supplementary file 3 — Additional file 3: Table S3. Production efficiency of the Salaya1 strain in each production line under laboratory and semi-mass rearing conditions. [file 12863_2020_933_MOESM3_ESM.pdf]

Additional file 3:

Table S3. Production efficiency of the Salaya1 strain in each production line under laboratory and semi-mass rearing conditions

**Parameter**	**Phayathai1 (wild-type strain)**	**Salaya1 (genetic sexing strain)**			
		**Clean stream**	**Initiation stream**	**Injection stream**	**Release stream**
Egg production (eggs/female/day)	n/a	39.84 ± 2.73 a	27.67 ± 3.34 b	23.18 ± 2.44 b	24.48 ± 2.23 b
Eggs reared under the laboratory conditions					
Fertility (%)	88.63 ± 0.47 a	49.41 ± 0.80 b	47.57 ± 1.01 b	47.06 ± 1.24 b	n/a
Pupal recovery (%)	89.25 ± 1.20 a	75.78 ± 0.98 b	76.70 ± 3.27 b	77.20 ± 1.17 b	n/a
Pupa weight (mg)	11.2 ± 0.64	n/a	n/a	n/a	n/a
Adult emergence (%)	77.64 ± 1.33 a	73.85 ± 4.65 a	73.91 ± 3.71 a	73.54 ± 3.67 a	n/a
Sex ratio (Male:Female)	1.03 ± 0.03 a	1.31 ± 0.02 b	1.32 ± 0.02 b	1.33 ± 0.03 b	0.98 ± 0.03 a*
Egg-to-adult recovery rate (%)	61.29 ± 1.24 a	27.48 ± 2.14 b	27.05 ± 2.59 b	26.73 ± 3.11 b	n/a
Eggs reared under the semi-mass rearing conditions					
Pupae production (%)	n/a	12.25 ± 0.62 a	11.50 ± 1.54 a	n/a	10.47 ± 1.19 a

Pupa weight (mg)	n/a	BP 11.00 ± 0.13,<br>WP 11.50 ± 0.18	BP 11.55 ± 0.28,<br>WP 11.77 ± 0.31	BP 11.17 ± 0.28,<br>WP 11.77 ± 0.31	BP 11.34 ± 0.13,<br>WP 11.75 ± 0.19
Egg-to-adult recovery rate (%)	n/a	9.07 ± 0.46 a	9.29 ± 0.92 a	n/a	8.45 ± 0.57 a

Mean ± standard error followed by the same letter are not significantly different from each other in the same parameter ($P < 0.05$) (Tukey HSD, $\alpha = 0.05$).

*Data from adults reared under semi-mass rearing conditions.
