# Supplementary material for: Genetic stability, genetic variation, and fitness performance of the genetic sexing Salaya1 strain for Bactrocera dorsalis, under long-term mass rearing conditions
Source: BMC Genet. 2020 Dec 18;21(Suppl 2):131. doi: 10.1186/s12863-020-00933-4 (PMC7747453; doi:10.1186/s12863-020-00933-4)
Supplement: Supplementary file 4 — Additional file 4: Figure S1. Results of ISSR_01 marker analysis. Figure S2. Results of ISSR_02 marker analysis. Figure S3. Results of ISSR_03 marker analysis. Figure S4. Results of ISSR_04 marker analysis. Figure S5. Results of ISSR_05 marker analysis. Figure S6. Results of ISSR_06 marker analysis. [file 12863_2020_933_MOESM4_ESM.pdf]

Additional file 4:

a)

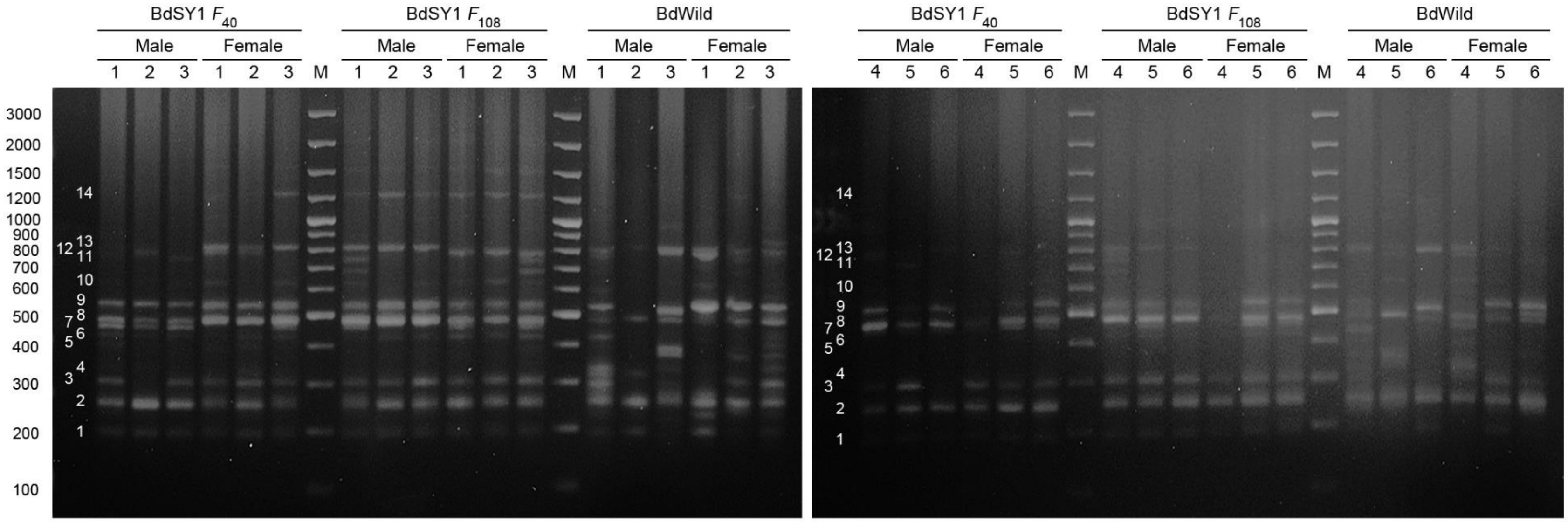

b)

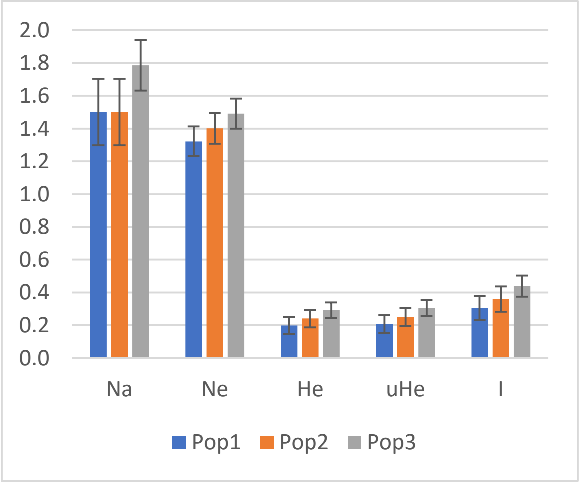

c)

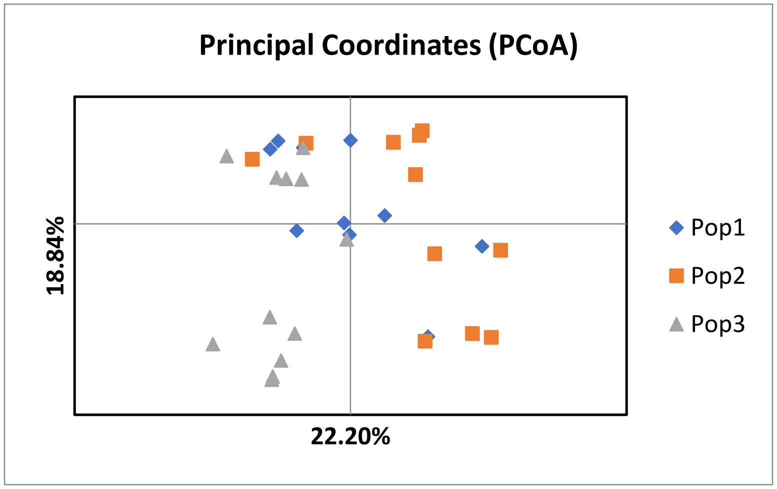

Fig. S1 Results of ISSR_01 marker analysis: a) Banding patterns among two generations of the clean stream and the wild population. No.1 to 14 represent the selected bands. M: 100 bp plus DNA ladder (100 to 3000 bp). b) Genetic variation estimated from 14 bands. Na: the number of alleles; Ne: the number of effective alleles; He: expected heterozygosity; I: Shannon's information index; %P: percentage of polymorphic loci. c) Principle coordinate analysis (PCoA). The planes of the first two principal coordinates explain 22.20% and 18.84% of total genetic variation, respectively. Pop1: the Salaya1 clean stream F_{40}; Pop2: the Salaya1 clean stream F_{108}; Pop3: the wild population.

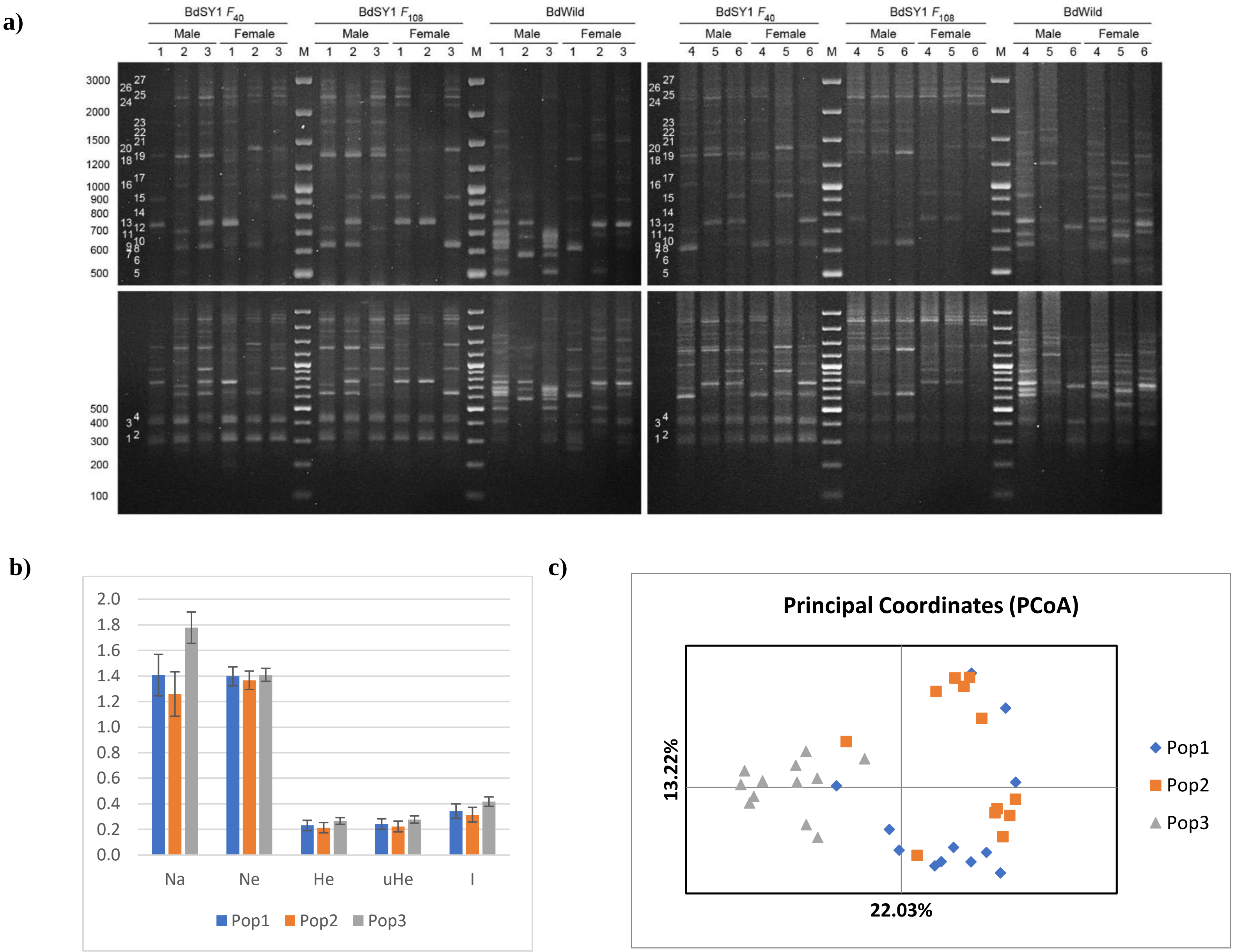
a)
BdSY1 F40
BdSY1 F108
BdWild
Male
Female
1 2 3 1 2 3 M 1 2 3 1 2 3 M 1 2 3 1 2 3
4 5 6 4 5 6 M 4 5 6 4 5 6 M 4 5 6 4 5 6
3000
2000
1500
1200
1000
900
800
700
600
500
400
300
200
100
b)
2.0
1.8
1.6
1.4
1.2
1.0
0.8
0.6
0.4
0.2
0.0
Na
Ne
He
uHe
I
Pop1
Pop2
Pop3
c)
Principal Coordinates (PCoA)
13.22%
22.03%
Pop1
Pop2
Pop3

Fig. S2 Results of ISSR_02 marker analysis: a) Banding patterns among two generations of the clean stream and the wild population. No.1 to 27 represent the selected bands. M: 100 bp plus DNA ladder (100 to 3000 bp). b) Genetic variation estimated from 27 bands. Na: the number of alleles; Ne: the number of effective alleles; He: expected heterozygosity; I: Shannon's information index; %P: percentage of polymorphic loci. c) Principle coordinate analysis (PCoA). The planes of the first two principal coordinates explain 22.03% and 13.22% of total genetic variation, respectively. Pop1: the Salaya1 clean stream F_{40}; Pop2: the Salaya1 clean stream F_{108}; Pop3: the wild population.

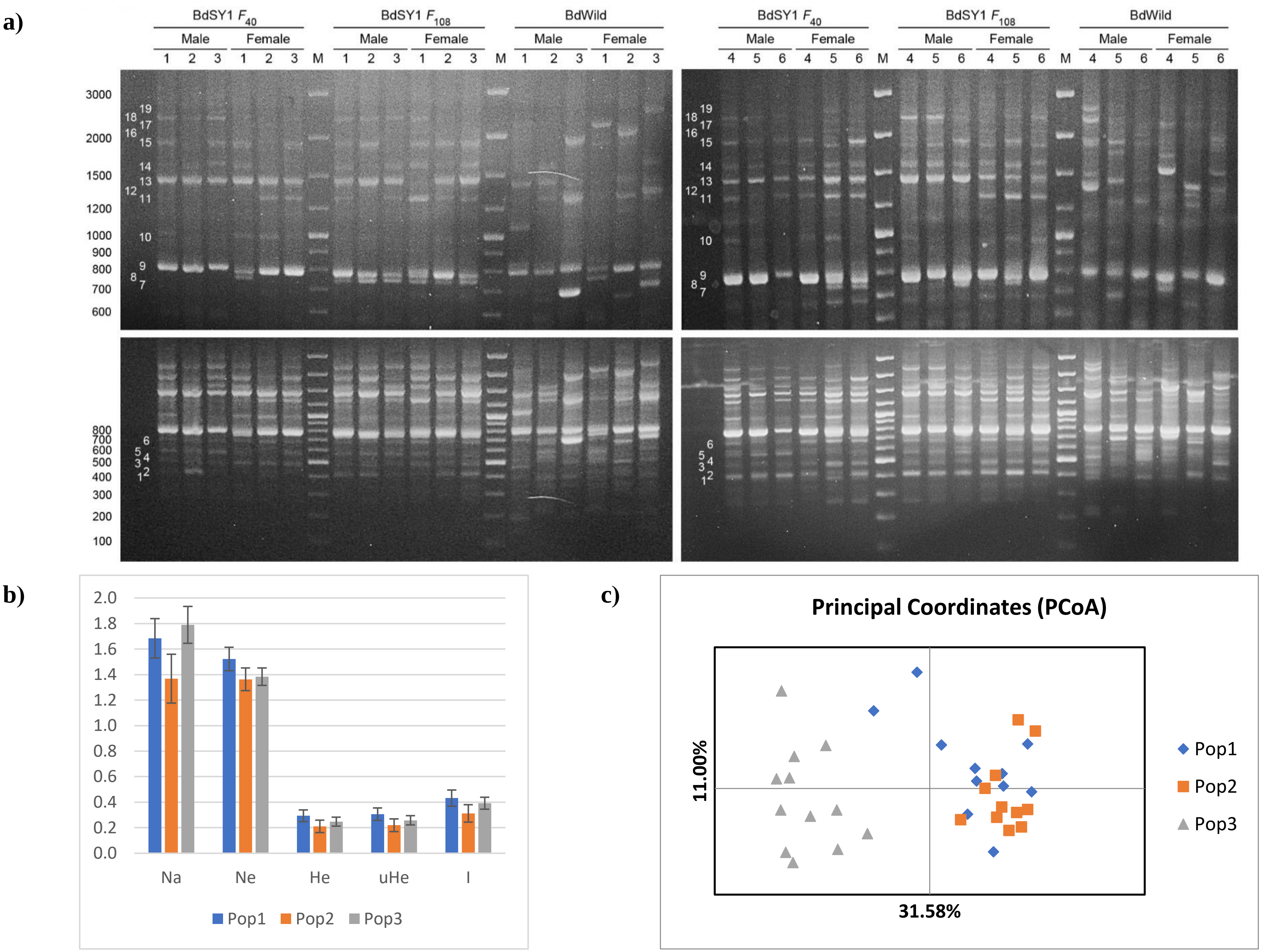

a)
BdSY1 F40
BdSY1 F108
BdWild
Male
Female
M
1 2 3
4 5 6
3000
2000
1500
1200
1000
900
800
700
600
500
400
300
200
100
b)
2.0
1.8
1.6
1.4
1.2
1.0
0.8
0.6
0.4
0.2
0.0
Na
Ne
He
uHe
I
Pop1
Pop2
Pop3
c)
Principal Coordinates (PCoA)
11.00%
31.58%
Pop1
Pop2
Pop3

Fig. S3 Results of ISSR_03 marker analysis: a) Banding patterns among two generations of the clean stream and the wild population. No.1 to 19 represent the selected bands. M: 100 bp plus DNA ladder (100 to 3000 bp). b) Genetic variation estimated from 19 bands. Na: the number of alleles; Ne: the number of effective alleles; He: expected heterozygosity; I: Shannon's information index; %P: percentage of polymorphic loci. c) Principle coordinate analysis (PCoA). The planes of the first two principal coordinates explain 31.58% and 11.00% of total genetic variation, respectively. Pop1: the Salaya1 clean stream F_{40}; Pop2: the Salaya1 clean stream F_{108}; Pop3: the wild population.

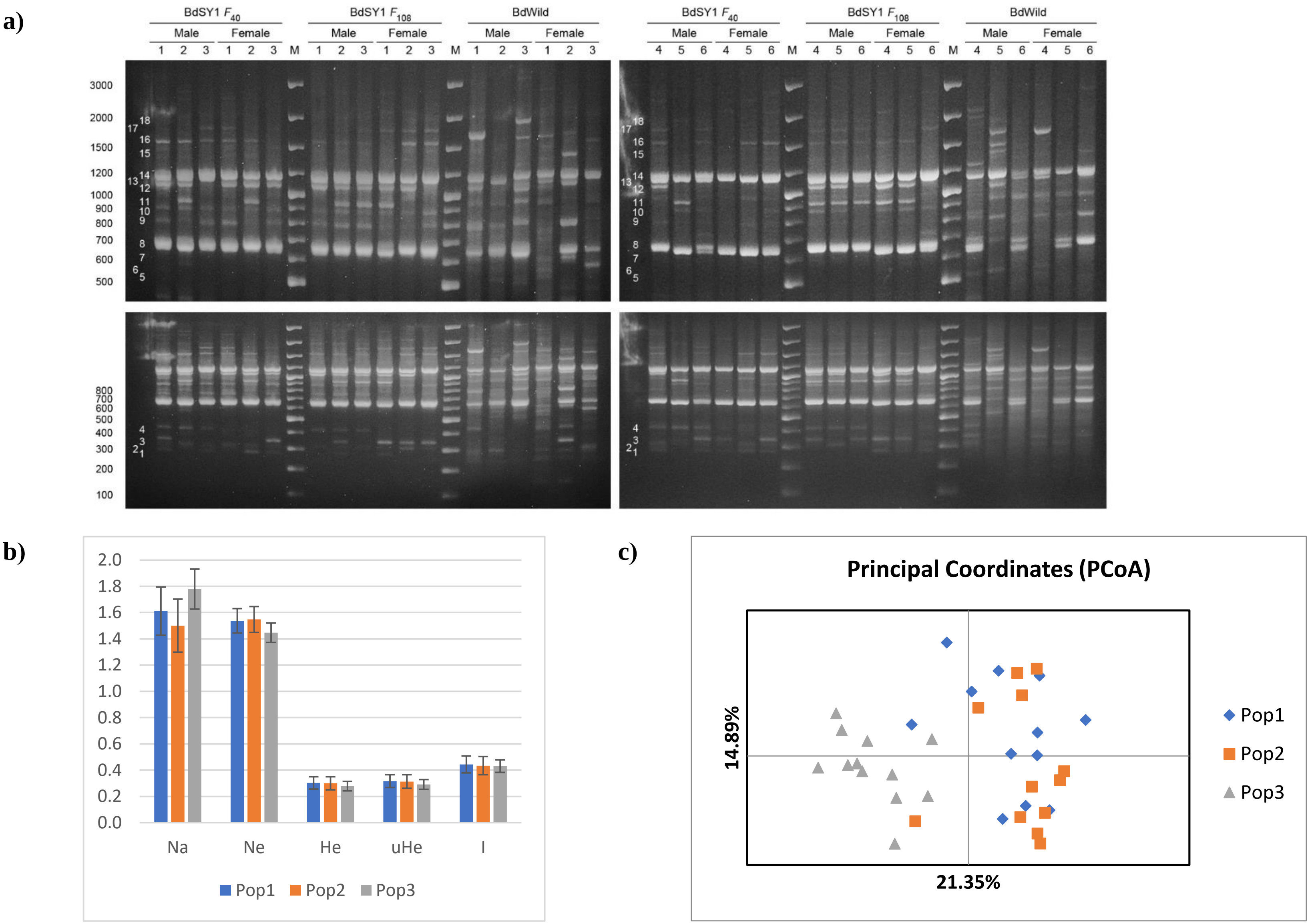
a)
BdSY1 F40
BdSY1 F108
BdWild
Male
Female
1 2 3 1 2 3 M
4 5 6 4 5 6 M
3000
2000
1500
1200
1000
900
800
700
600
500
400
300
200
100
b)
2.0
1.8
1.6
1.4
1.2
1.0
0.8
0.6
0.4
0.2
0.0
Na
Ne
He
uHe
I
Pop1
Pop2
Pop3
c)
Principal Coordinates (PCoA)
14.89%
21.35%
Pop1
Pop2
Pop3

Fig. S4 Results of ISSR_04 marker analysis: a) Banding patterns among two generations of the clean streams and the wild population. No.1 to 18 represent the selected bands. M: 100 bp plus DNA ladder (100 to 3000 bp). b) Genetic variation estimated from 18 bands. Na: the number of alleles; Ne: the number of effective alleles; He: expected heterozygosity; I: Shannon's information index; %P: percentage of polymorphic loci. c) Principle coordinate analysis (PCoA). The planes of the first two principal coordinates explain 21.35% and 14.89% of total genetic variation, respectively. Pop1: the Salaya1 clean stream F_{40}; Pop2: the Salaya1 clean stream F_{108}; Pop3: the wild population.

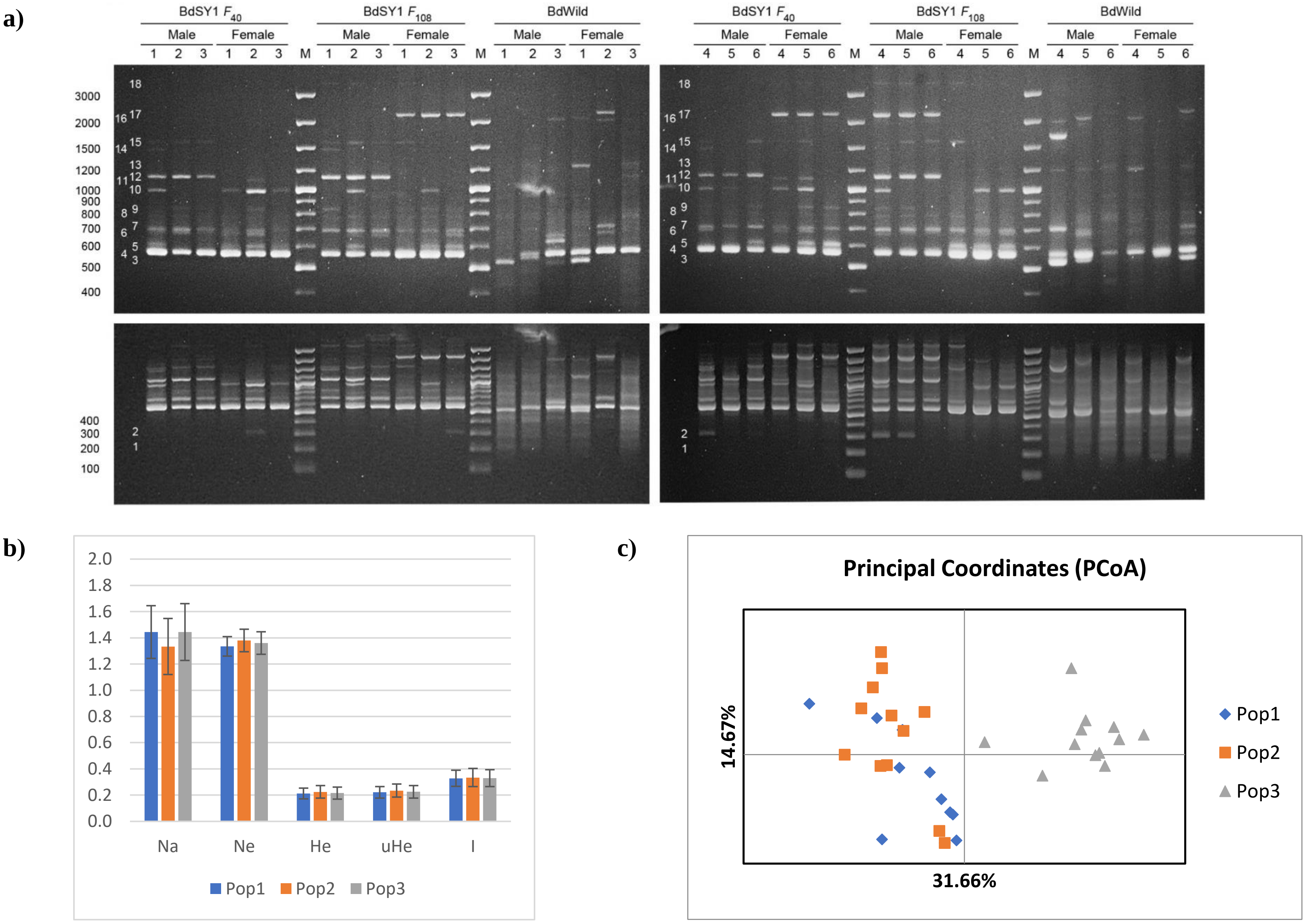
a)
BdSY1 F40
BdSY1 F108
BdWild
Male
Female
M
3000
2000
1500
1200
1000
900
800
700
600
500
400
300
200
100
b)
2.0
1.8
1.6
1.4
1.2
1.0
0.8
0.6
0.4
0.2
0.0
Na
Ne
He
uHe
I
Pop1
Pop2
Pop3
c)
Principal Coordinates (PCoA)
14.67%
31.66%
Pop1
Pop2
Pop3

Fig. S5 Results of ISSR_05 marker analysis: a) Banding patterns among two generations of the clean stream and the wild population. No.1 to 18 represent the selected bands. M: 100 bp plus DNA ladder (100 to 3000 bp). b) Genetic variation estimated from 18 bands. Na: the number of alleles; Ne: the number of effective alleles; He: expected heterozygosity; I: Shannon's information index; %P: percentage of polymorphic loci. c) Principle coordinate analysis (PCoA). The planes of the first two principal coordinates explain 31.66% and 14.67% of total genetic variation, respectively. Pop1: the Salaya1 clean stream F_{40}; Pop2: the Salaya1 clean stream F_{108}; Pop3: the wild population.

a)

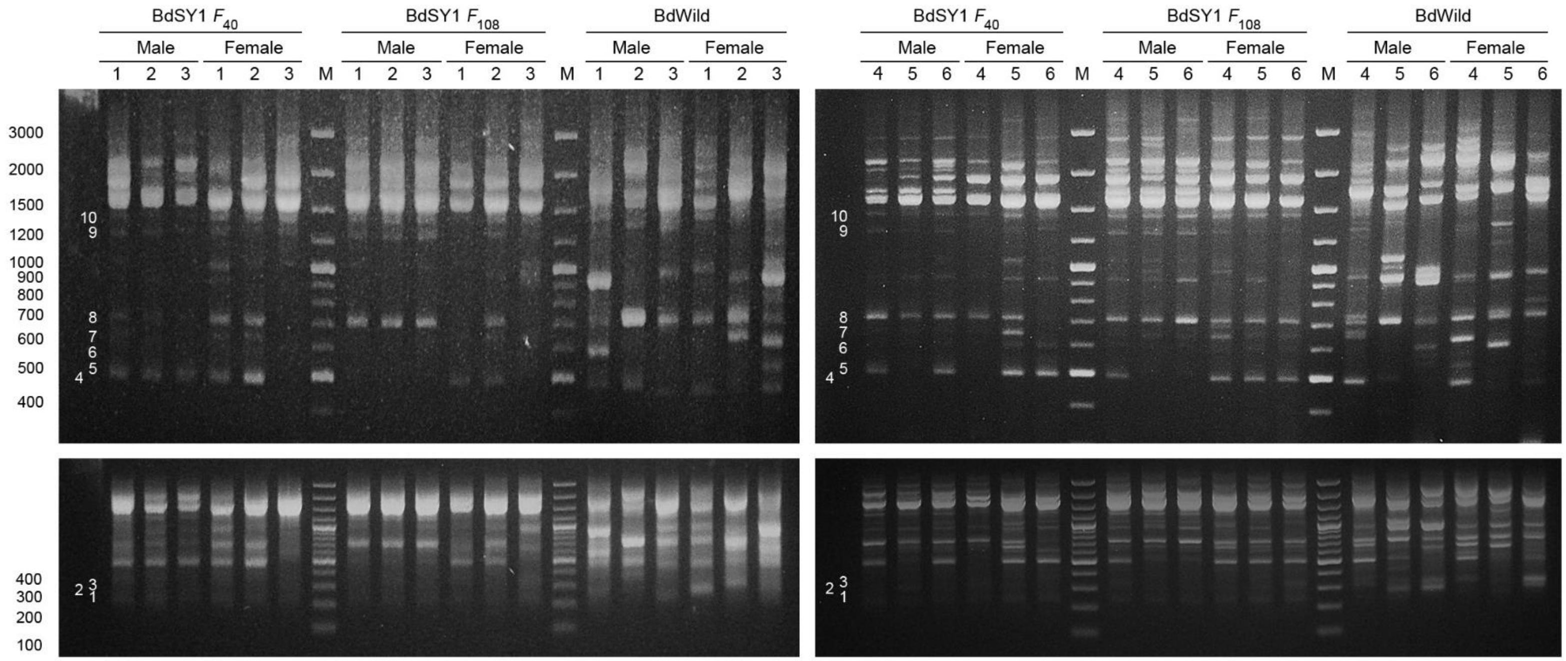

BdSY1 F_{40}
BdSY1 F_{108}
BdWild
Male
Female
1 2 3 1 2 3 M 1 2 3 1 2 3 M 1 2 3 1 2 3
4 5 6 4 5 6 M 4 5 6 4 5 6 M 4 5 6 4 5 6
3000
2000
1500
1200
1000
900
800
700
600
500
400
300
200
100
10
9
8
7
6
5
4
3
2
1

b)

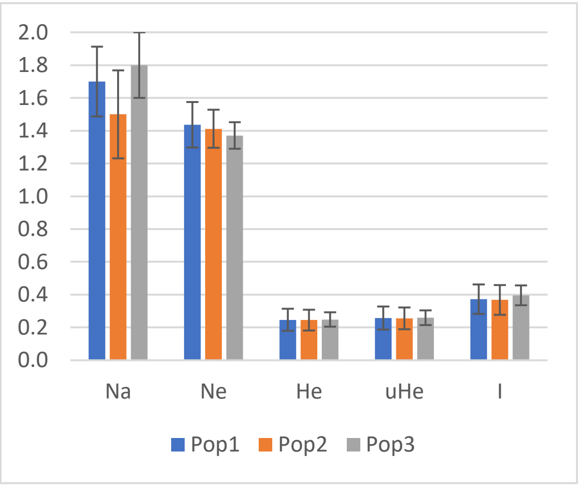

2.0
1.8
1.6
1.4
1.2
1.0
0.8
0.6
0.4
0.2
0.0
Na
Ne
He
uHe
I
Pop1
Pop2
Pop3

c)

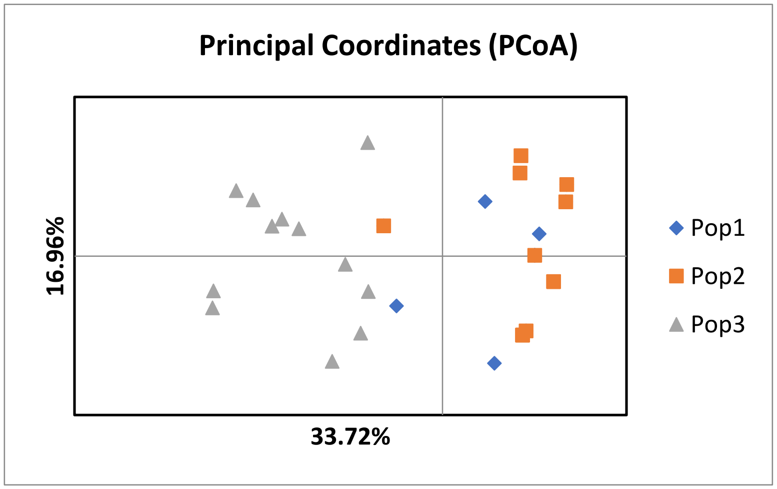

Principal Coordinates (PCoA)
16.96%
33.72%
Pop1
Pop2
Pop3

Fig. S6 Results of ISSR_06 marker analysis: a) Banding patterns among two generations of the clean stream and the wild population. No. 1 to 10 represent the selected bands. M: 100 bp plus DNA ladder (100 to 3000 bp). b) Genetic variation estimated from 10 bands. Na: the number of alleles; Ne: the number of effective alleles; He: expected heterozygosity; I: Shannon's information index; %P: percentage of polymorphic loci. c) Principle coordinate analysis (PCoA). The planes of the first two principal coordinates explain 33.72% and 16.96% of total genetic variation, respectively. Pop1: the Salaya1 clean stream F_{40}; Pop2: the Salaya1 clean stream F_{108}; Pop3: the wild population.
