# Supplementary material for: Genetic stability, genetic variation, and fitness performance of the genetic sexing Salaya1 strain for Bactrocera dorsalis, under long-term mass rearing conditions
Source: BMC Genet. 2020 Dec 18;21(Suppl 2):131. doi: 10.1186/s12863-020-00933-4 (PMC7747453; doi:10.1186/s12863-020-00933-4)
Supplement: Supplementary file 5 — Additional file 5: Table S4. Six polymorphic ISSR primers and their matrices of obtained band sizes. [file 12863_2020_933_MOESM5_ESM.pdf]

Additional file 5:

Table S4. Six polymorphic ISSR primers and their matrices of obtained band sizes

No.	Primer	Sequence (5'→3')	(%P)	No. of selected bands	Approximate amplicon size (bp) estimated from electrophoretic mobility plots
1.	ISSR_01	$(GT)_8AG$	71.43%	14	187, 230, 281, 304, 384, 407, 464, 473, 535, 634, 775, 810, 849, 1321
2.	ISSR_02	$(GT)_8AA$	69.17%	27	289, 319, 396, 417, 491, 545, 568, 608, 613, 643, 683, 721, 759, 831, 948, 1062, 1145, 1323, 1400, 1492, 1590, 1705, 1895, 2228, 2347, 2555, 2732
3.	ISSR_03	$(AG)_8C$	75.44%	19	374, 405, 467, 502, 589, 687, 714, 742, 804, 1000, 1323, 1399, 1492, 1677, 1969, 2124, 2261, 2366, 2516
4.	ISSR_04	$(AG)_8G$	79.63%	18	248, 284, 313, 404, 503, 531, 592, 663, 821, 886, 969, 1095, 1152, 1229, 1472, 1659, 1853, 1973
5.	ISSR_05	$(AG)_8AA$	66.67%	18	200, 306, 518, 555, 596, 670, 719, 809, 866, 1035, 1146, 1181, 1322, 1557, 1664, 2097, 2187, 3002
6.	ISSR_06	$(GTT)_8C$	80.00%	10	209, 271, 337,479, 531, 582, 644,719, 1310, 1479
Average	-	-	72.96%	17.6	-

%P: Percentage of polymorphism, bp: base pairs
