# Supplementary material for: Genetic stability, genetic variation, and fitness performance of the genetic sexing Salaya1 strain for Bactrocera dorsalis, under long-term mass rearing conditions
Source: BMC Genet. 2020 Dec 18;21(Suppl 2):131. doi: 10.1186/s12863-020-00933-4 (PMC7747453; doi:10.1186/s12863-020-00933-4)
Supplement: Supplementary file 7 — Additional file 7: Figure S7. Raw gel images from ISSR_01 marker analysis. Figure S8. Raw gel images from ISSR_02 marker analysis. Figure S9. Raw gel images from ISSR_03 marker analysis. Figure S10. Raw gel images from ISSR_04 marker analysis. Figure S11. Raw gel images from ISSR_05 marker analysis. Figure S12. Raw gel images from ISSR_06 marker analysis. [file 12863_2020_933_MOESM7_ESM.pdf]

Additional file 7:

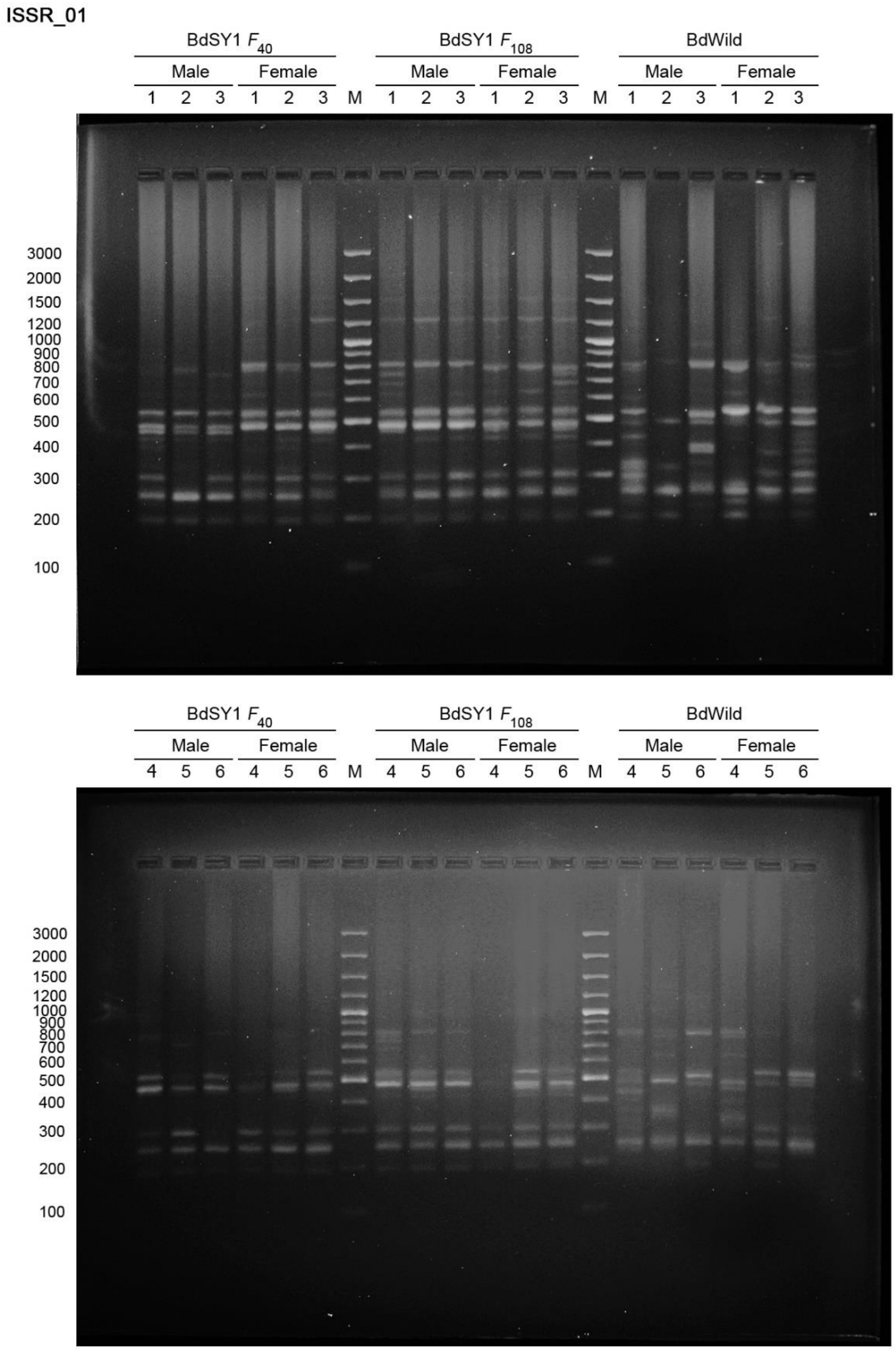

Fig. S7 Raw gel images from ISSR_01 marker analysis. These gel images were cropped and represented in Additional file 4: Fig. S1a. Banding patterns among two generations of the clean stream and the wild population were visualized on 2% agarose gel electrophoresis, at 70 V for 2 hours. M: 100 bp plus DNA ladder (100 to 3000 bp). Pop1: the Salaya1 clean stream F_{40}; Pop2: the Salaya1 clean stream F_{108}; Pop3: the wild population.

a) ISSR_02

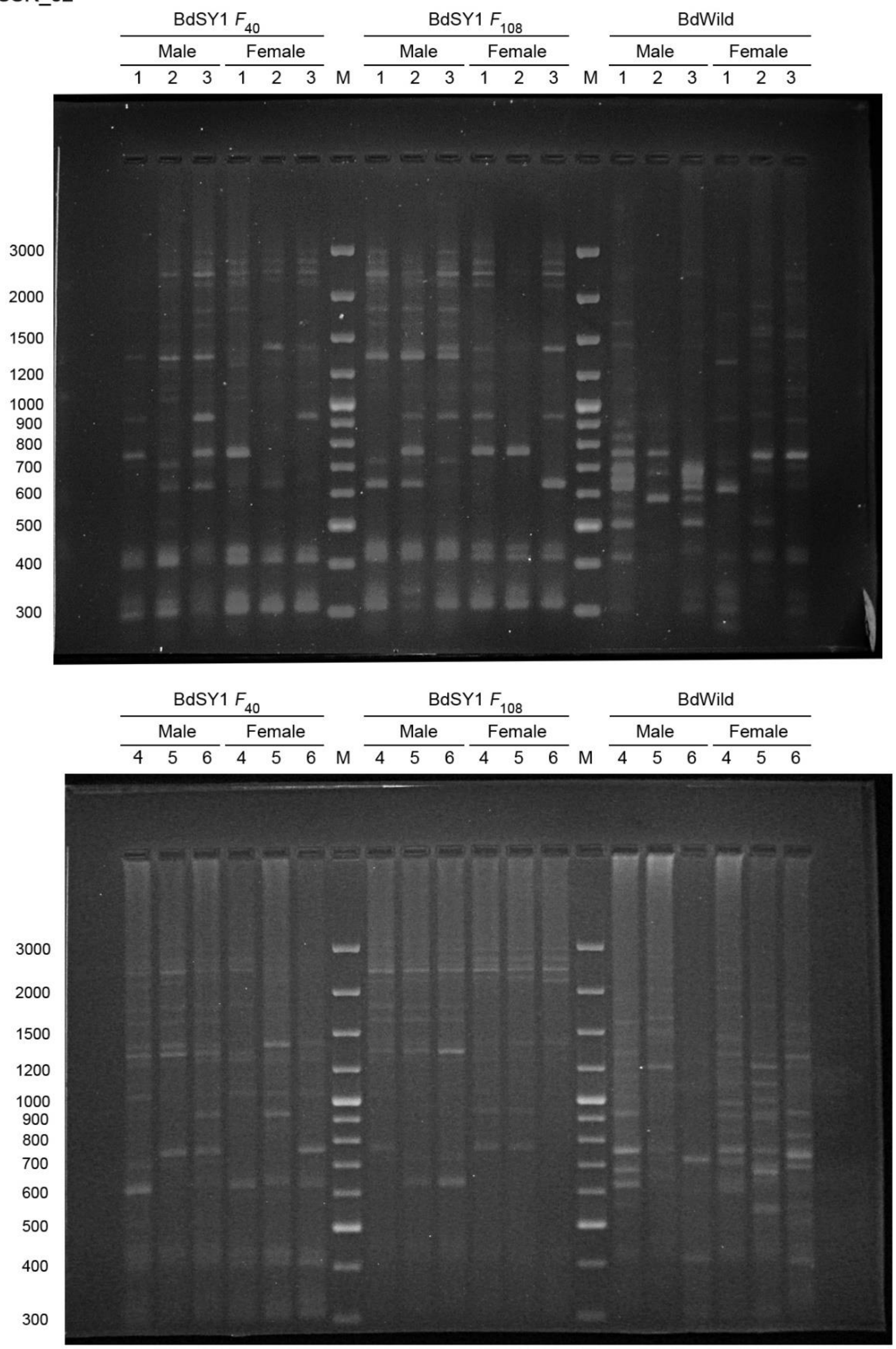

BdSY1 F_{40}
BdSY1 F_{108}
BdWild
Male
Female
Male
Female
Male
Female
1 2 3 1 2 3 M 1 2 3 1 2 3 M 1 2 3 1 2 3
3000
2000
1500
1200
1000
900
800
700
600
500
400
300
BdSY1 F_{40}
BdSY1 F_{108}
BdWild
Male
Female
Male
Female
Male
Female
4 5 6 4 5 6 M 4 5 6 4 5 6 M 4 5 6 4 5 6
3000
2000
1500
1200
1000
900
800
700
600
500
400
300

b) ISSR_02

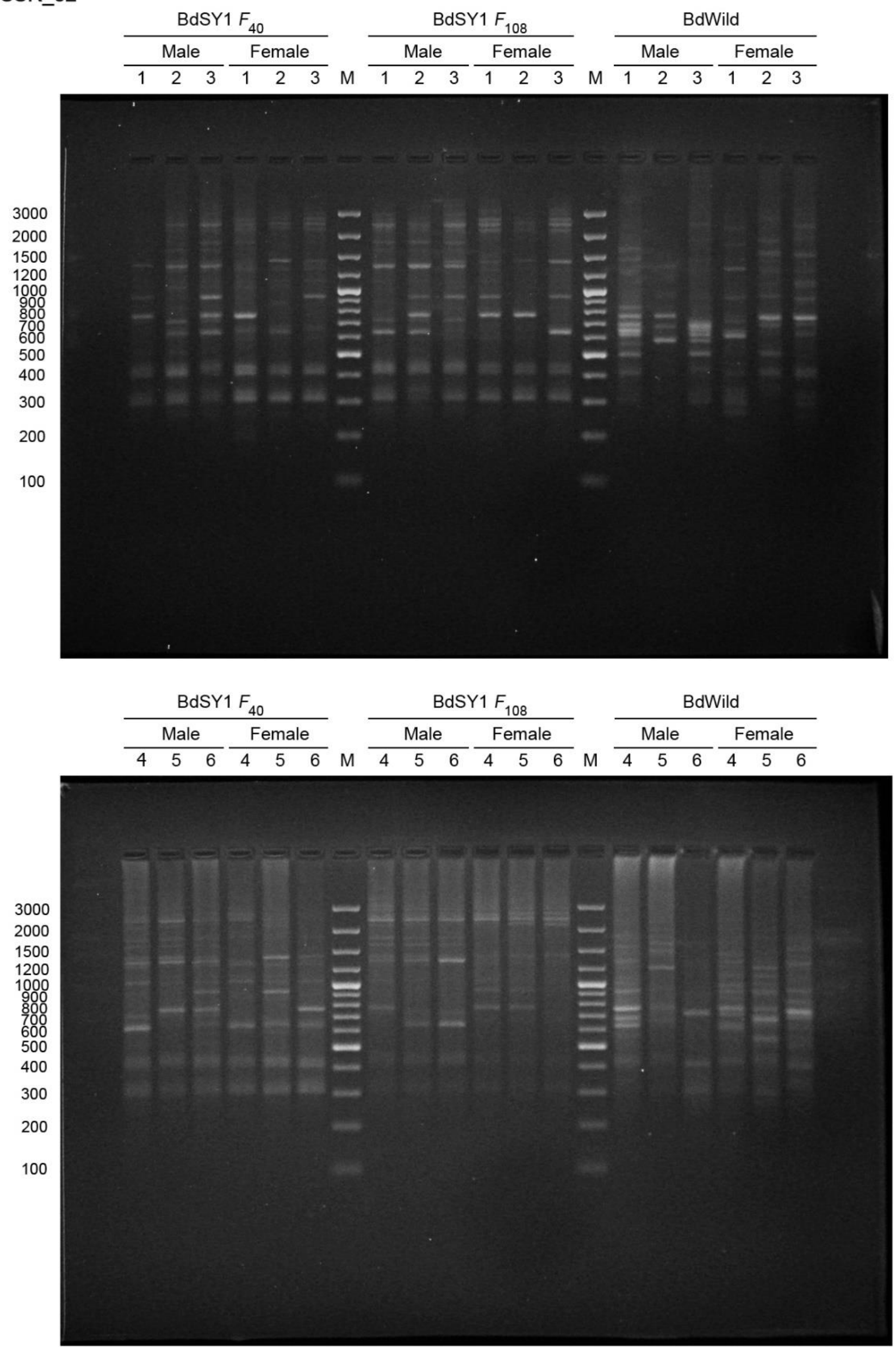

BdSY1 F40
BdSY1 F108
BdWild
Male
Female
Male
Female
Male
Female
1 2 3 1 2 3 M 1 2 3 1 2 3 M 1 2 3 1 2 3
3000
2000
1500
1200
1000
900
800
700
600
500
400
300
200
100
BdSY1 F40
BdSY1 F108
BdWild
Male
Female
Male
Female
Male
Female
4 5 6 4 5 6 M 4 5 6 4 5 6 M 4 5 6 4 5 6
3000
2000
1500
1200
1000
900
800
700
600
500
400
300
200
100

Fig. S8 Raw gel images from ISSR_02 marker analysis. a) These gel images were cropped and represented in the upper panel of Additional file 4: Fig. S2a. Banding patterns among two generations of the clean stream and the wild population were visualized on 2% agarose gel electrophoresis, at 50 V for 5 hours. b) These gel images were cropped and represented in the lower panel of Additional file 4: Fig. S2a. Banding patterns among two generations of the clean stream and the wild population were visualized on 2% agarose gel electrophoresis, at 50 V for 2 hours. M: 100 bp plus DNA ladder (100 to 3000 bp). Pop1: the Salaya1 clean stream F_{40}; Pop2: the Salaya1 clean stream F_{108}; Pop3: the wild population.

a) **ISSR_03**

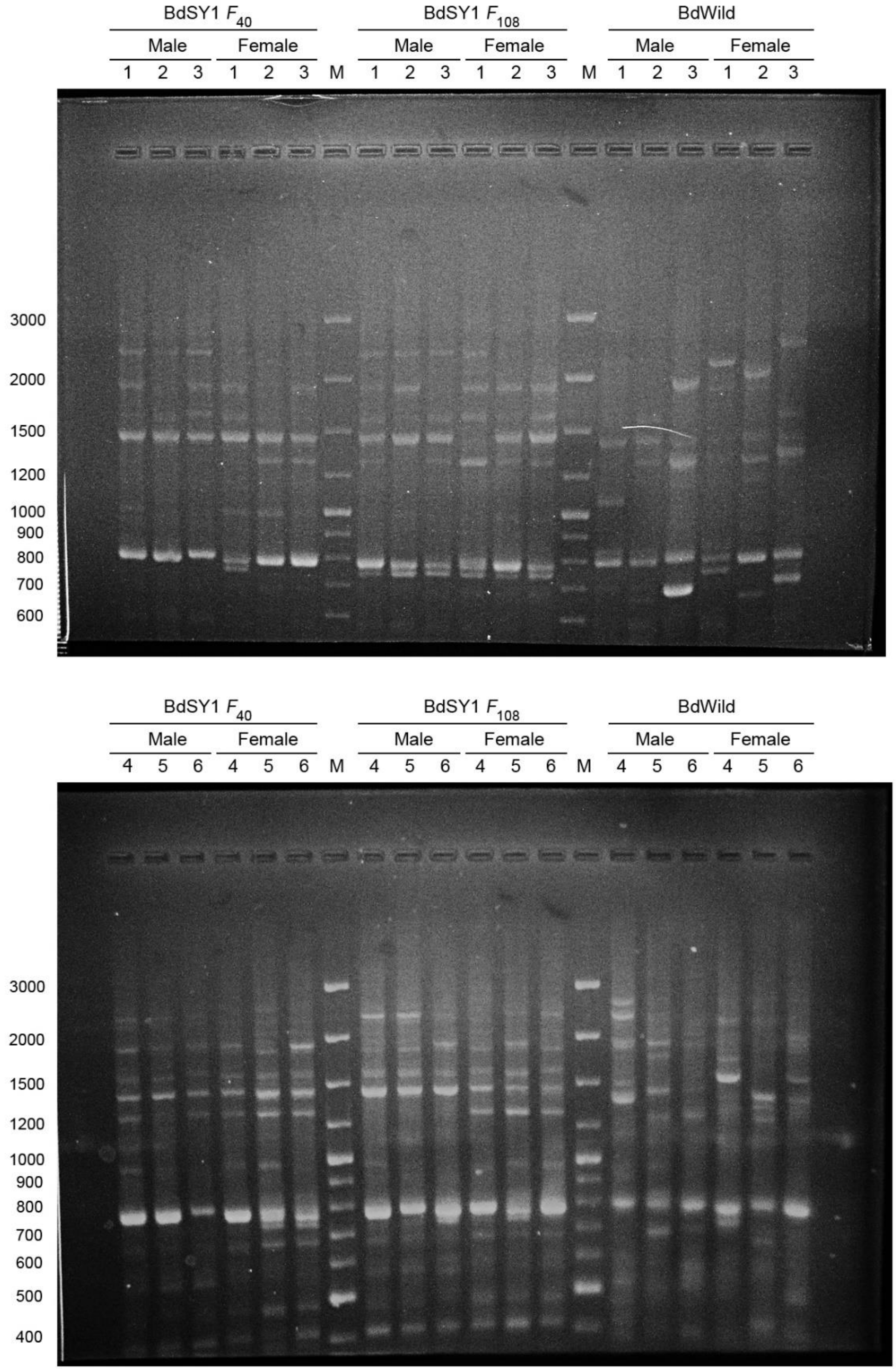
BdSY1 F40
BdSY1 F108
BdWild
Male
Female
Male
Female
Male
Female
1 2 3 1 2 3 M 1 2 3 1 2 3 M 1 2 3 1 2 3
3000
2000
1500
1200
1000
900
800
700
600
BdSY1 F40
BdSY1 F108
BdWild
Male
Female
Male
Female
Male
Female
4 5 6 4 5 6 M 4 5 6 4 5 6 M 4 5 6 4 5 6
3000
2000
1500
1200
1000
900
800
700
600
500
400

b) ISSR_03

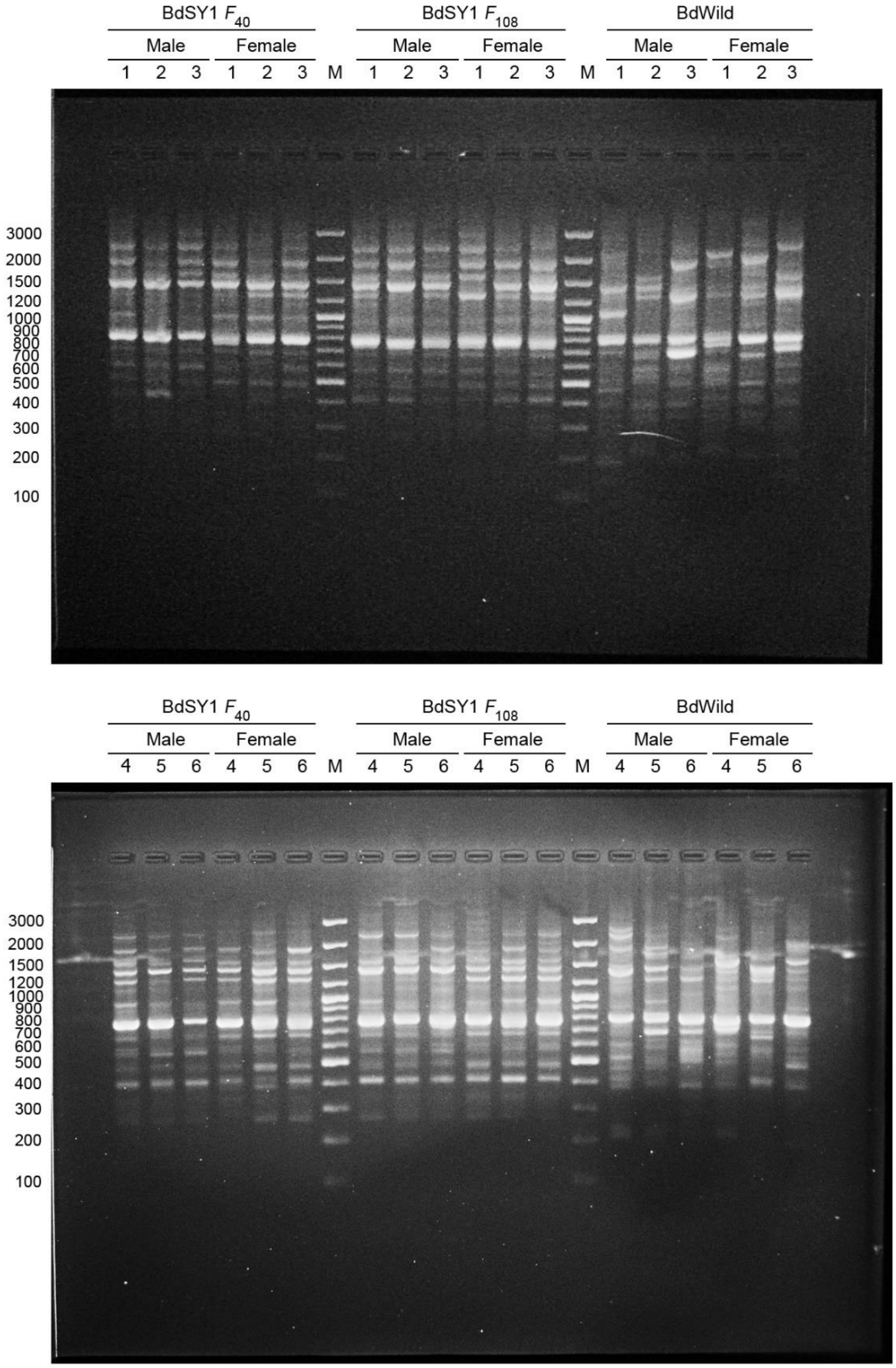
BdSY1 F_{40}
BdSY1 F_{108}
BdWild
Male
Female
1 2 3 1 2 3 M 1 2 3 1 2 3 M 1 2 3 1 2 3
3000
2000
1500
1200
1000
900
800
700
600
500
400
300
200
100
4 5 6 4 5 6 M 4 5 6 4 5 6 M 4 5 6 4 5 6

Fig. S9 Raw gel images from ISSR_03 marker analysis. a) These gel images were cropped and represented in the upper panel of Additional file 4: Fig. S3a. Banding patterns among two generations of the clean stream and the wild population were visualized on 2% agarose gel electrophoresis, at 70 V for 4 hours. b) These gel images were cropped and represented in the lower panel of Additional file 4: Fig. S3a. Banding patterns among two generations of the clean stream and the wild population were visualized on 2% agarose gel electrophoresis, at 70 V for 2 hours. M: 100 bp plus DNA ladder (100 to 3000 bp). Pop1: the Salaya1 clean stream F_{40}; Pop2: the Salaya1 clean stream F_{108}; Pop3: the wild population.

a) ISSR_04

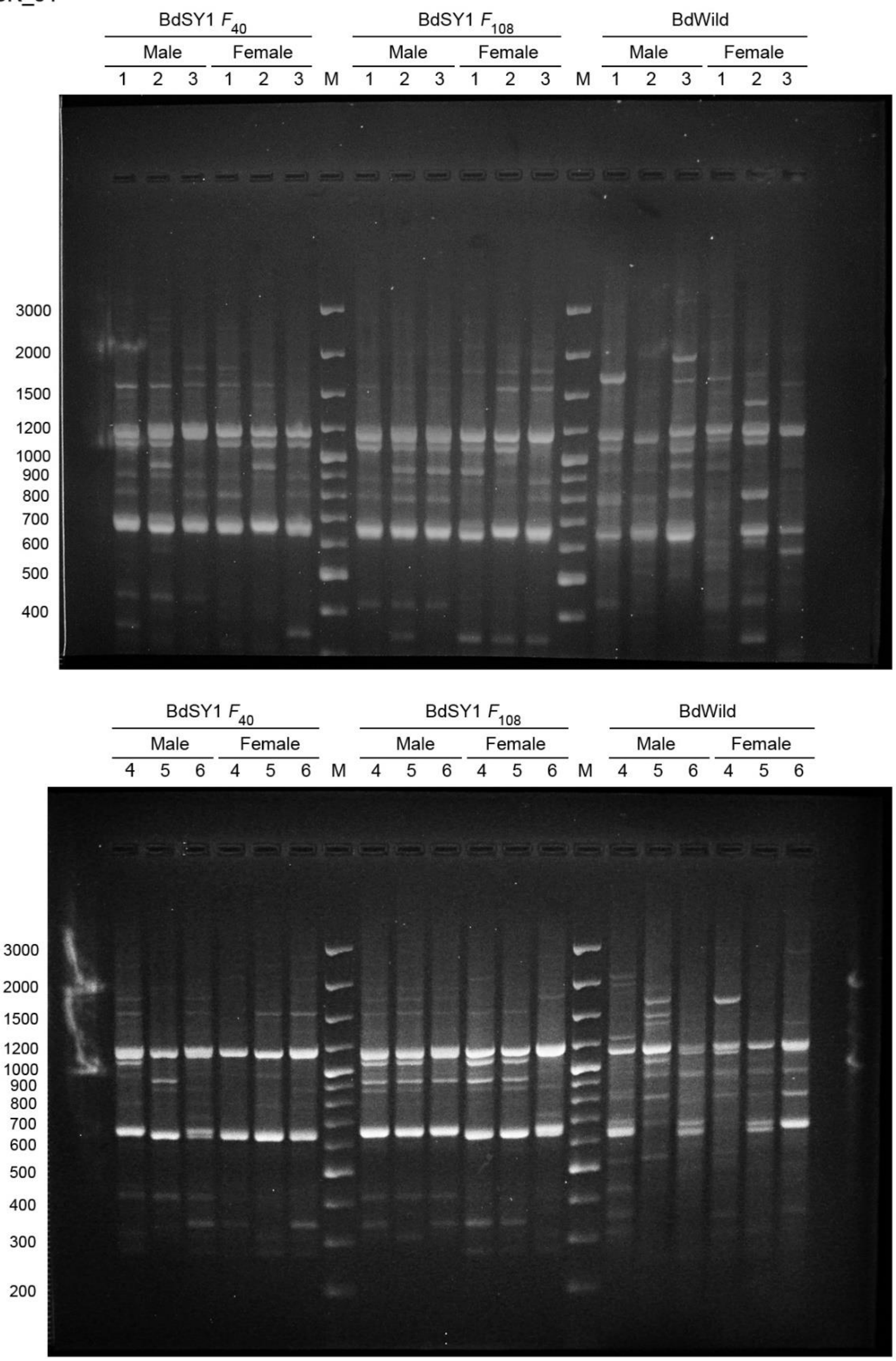
BdSY1 F_{40}
BdSY1 F_{108}
BdWild
Male
Female
Male
Female
Male
Female
1 2 3 1 2 3 M 1 2 3 1 2 3 M 1 2 3 1 2 3
3000
2000
1500
1200
1000
900
800
700
600
500
400
BdSY1 F_{40}
BdSY1 F_{108}
BdWild
Male
Female
Male
Female
Male
Female
4 5 6 4 5 6 M 4 5 6 4 5 6 M 4 5 6 4 5 6
3000
2000
1500
1200
1000
900
800
700
600
500
400
300
200

b) ISSR_04

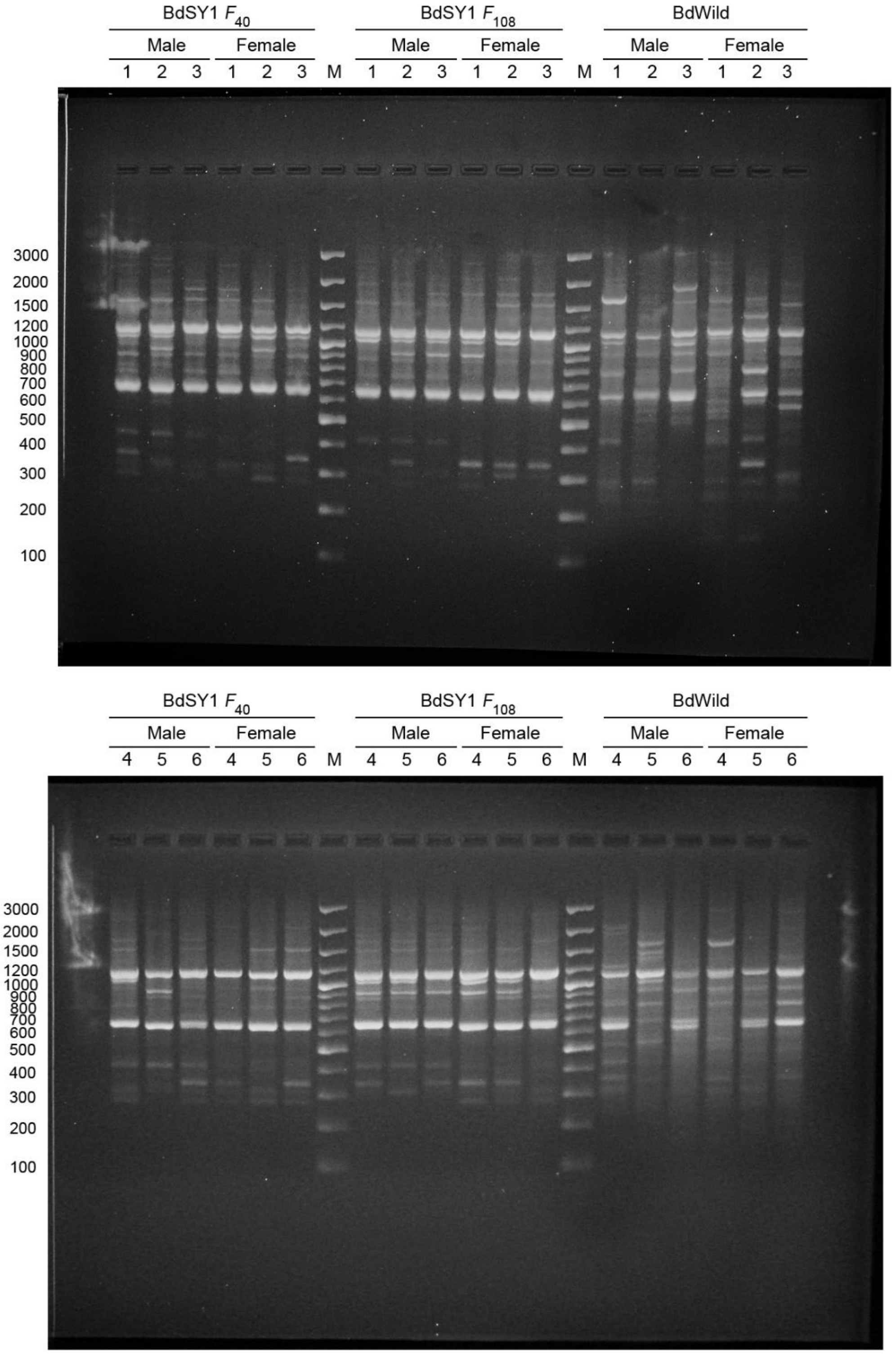
BdSY1 F_{40}
BdSY1 F_{108}
BdWild
Male
Female
1 2 3 1 2 3 M 1 2 3 1 2 3 M 1 2 3 1 2 3
3000
2000
1500
1200
1000
900
800
700
600
500
400
300
200
100
4 5 6 4 5 6 M 4 5 6 4 5 6 M 4 5 6 4 5 6

Fig. S10 Raw gel images from ISSR_04 marker analysis. a) These gel images were cropped and represented in the upper panel of Additional file 4: Fig. S4a. Banding patterns among two generations of the clean stream and the wild population were visualized on 2% agarose gel electrophoresis, at 60 V for 3 hours. b) These gel images were cropped and represented in the lower panel of Additional file 4: Fig. S4a. Banding patterns among two generations of the clean stream and the wild population were visualized on 2% agarose gel electrophoresis, at 60 V for 2 hours. M: 100 bp plus DNA ladder (100 to 3000 bp). Pop1: the Salaya1 clean stream F_{40}; Pop2: the Salaya1 clean stream F_{108}; Pop3: the wild population.

a) ISSR_05

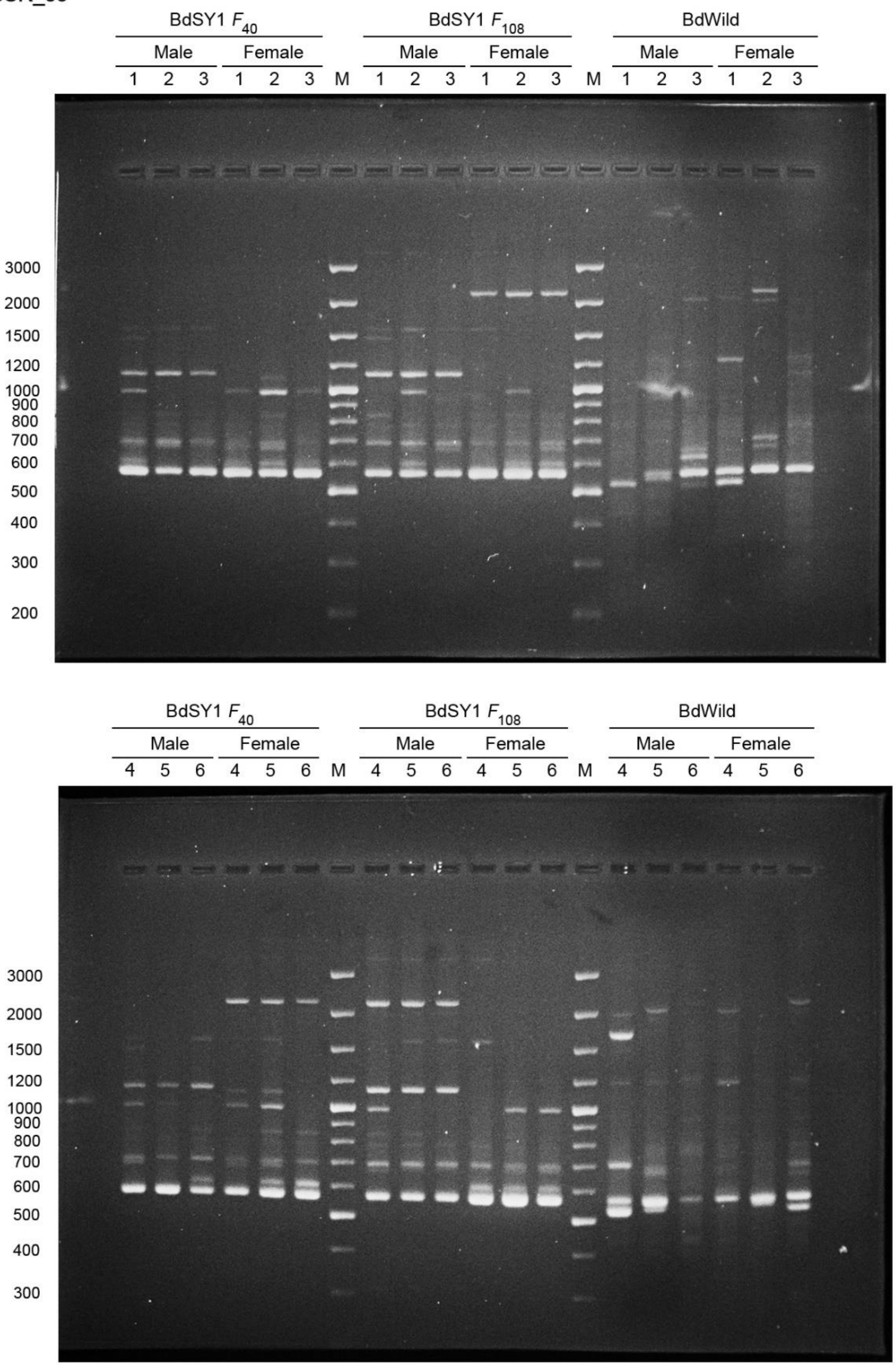

BdSY1 F40
BdSY1 F108
BdWild
Male
Female
1 2 3 1 2 3 M 1 2 3 1 2 3 M 1 2 3 1 2 3
3000
2000
1500
1200
1000
900
800
700
600
500
400
300
200
BdSY1 F40
BdSY1 F108
BdWild
Male
Female
4 5 6 4 5 6 M 4 5 6 4 5 6 M 4 5 6 4 5 6
3000
2000
1500
1200
1000
900
800
700
600
500
400
300

b) ISSR_05

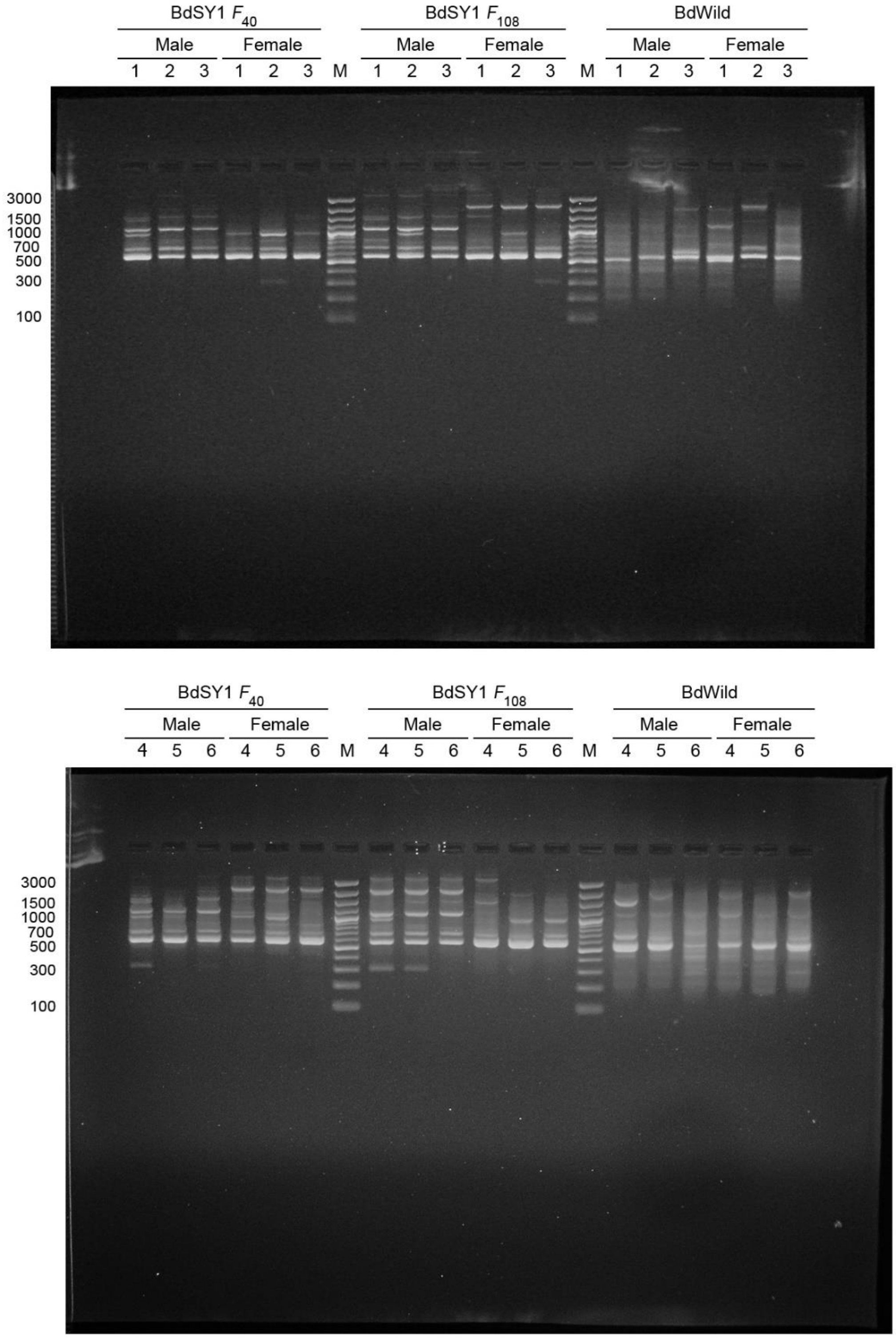

BdSY1 F_{40}
BdSY1 F_{108}
BdWild
Male
Female
1 2 3 1 2 3 M 1 2 3 1 2 3 M 1 2 3 1 2 3
3000
1500
1000
700
500
300
100
BdSY1 F_{40}
BdSY1 F_{108}
BdWild
Male
Female
4 5 6 4 5 6 M 4 5 6 4 5 6 M 4 5 6 4 5 6
3000
1500
1000
700
500
300
100

Fig. S11 Raw gel images from ISSR_05 marker analysis. a) These gel images were cropped and represented in the upper panel of Additional file 4: Fig. S5a. Banding patterns among two generations of the clean stream and the wild population were visualized on 2% agarose gel electrophoresis, at 60 V for 3 hours. b) These gel images were cropped and represented in the lower panel of Additional file 4: Fig. S5a. Banding patterns among two generations of the clean stream and the wild population were visualized on 2% agarose gel electrophoresis, at 60 V for 1 hour. M: 100 bp plus DNA ladder (100 to 3000 bp). Pop1: the Salaya1 clean stream F_{40}; Pop2: the Salaya1 clean stream F_{108}; Pop3: the wild population.

a) ISSR_06

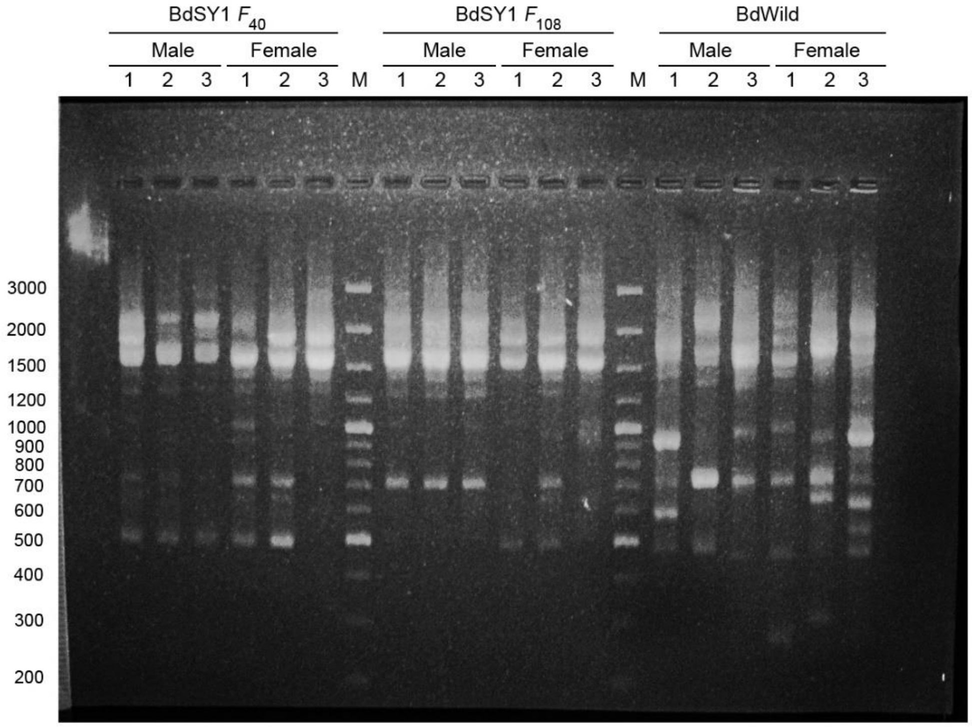
BdSY1 F_{40}
BdSY1 F_{108}
BdWild
Male Female M Male Female M Male Female
1 2 3 1 2 3 M 1 2 3 1 2 3 M 1 2 3 1 2 3
3000
2000
1500
1200
1000
900
800
700
600
500
400
300
200

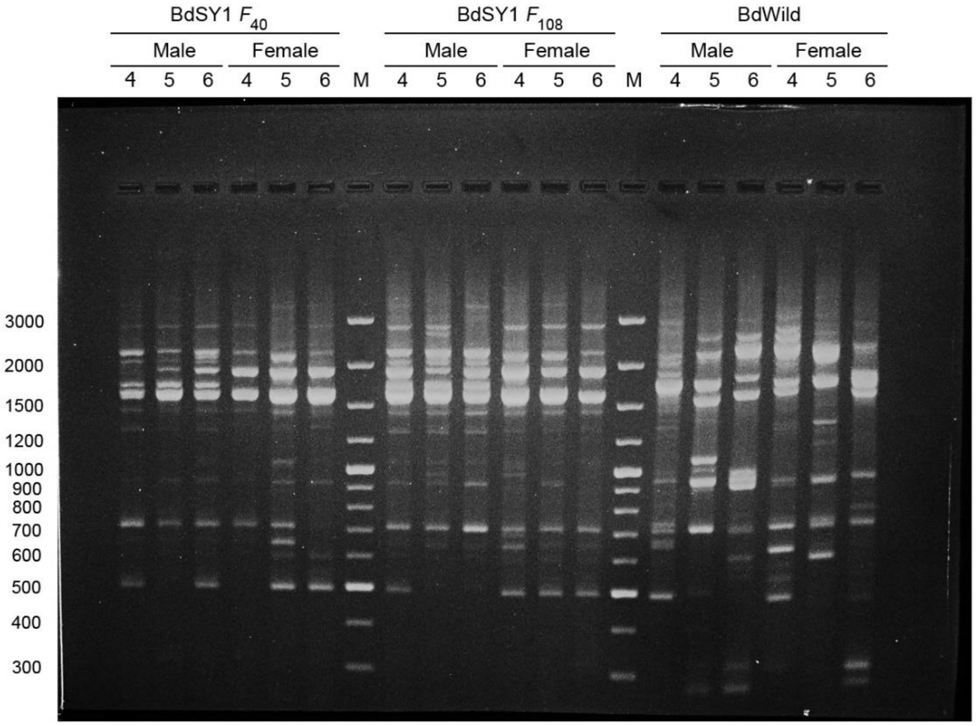
BdSY1 F_{40}
BdSY1 F_{108}
BdWild
Male Female M Male Female M Male Female
4 5 6 4 5 6 M 4 5 6 4 5 6 M 4 5 6 4 5 6
3000
2000
1500
1200
1000
900
800
700
600
500
400
300

b) ISSR_06

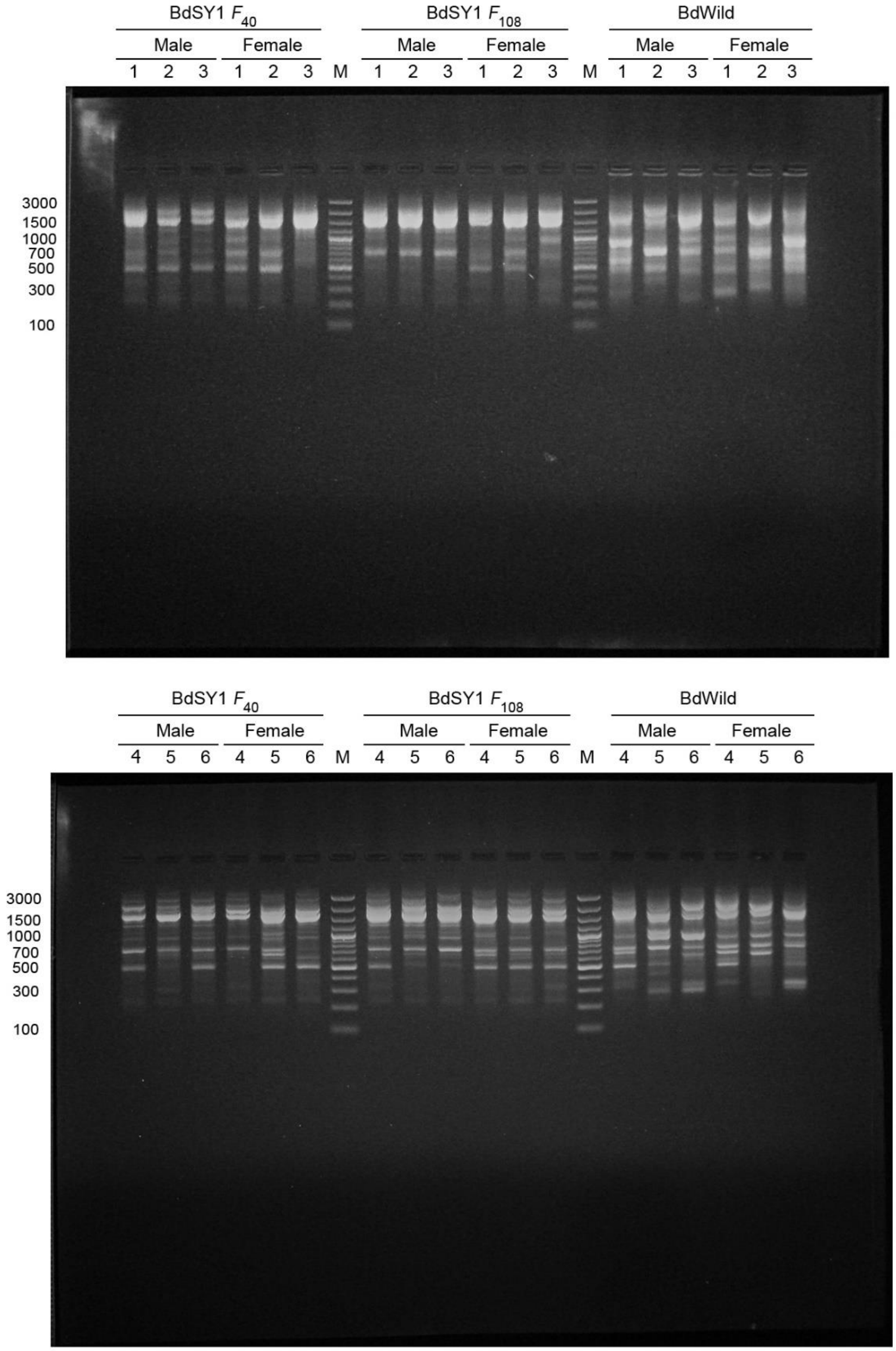

BdSY1 F_{40}
BdSY1 F_{108}
BdWild
Male
Female
1 2 3 1 2 3 M 1 2 3 1 2 3 M 1 2 3 1 2 3
3000
1500
1000
700
500
300
100
4 5 6 4 5 6 M 4 5 6 4 5 6 M 4 5 6 4 5 6

Fig. S12 Raw gel images from ISSR_06 marker analysis. a) These gel images were cropped and represented in the upper panel of Additional file 4: Fig. S6a. Banding patterns among two generations of the clean stream and the wild population were visualized on 2% agarose gel electrophoresis, at 60 V for 3 hours. b) These gel images were cropped and represented in the lower panel of Additional file 4: Fig. S6a. Banding patterns among two generations of the clean stream and the wild population were visualized on 2% agarose gel electrophoresis, at 60 V for 1 hour. M: 100 bp plus DNA ladder (100 to 3000 bp). Pop1: the Salaya1 clean stream F_{40}; Pop2: the Salaya1 clean stream F_{108}; Pop3: the wild population.
